# Kinetic Phenomena in Mechanochemical Depolymerization of Poly(styrene)

**DOI:** 10.1021/acssuschemeng.3c05296

**Published:** 2023-12-16

**Authors:** Yuchen Chang, Sylvie J. Blanton, Ralph Andraos, Van Son Nguyen, Charles L. Liotta, F. Joseph Schork, Carsten Sievers

**Affiliations:** † School of Chemical & Biomolecular Engineering, Georgia Institute of Technology, Atlanta, Georgia 30332, United States; ‡ Department of Chemistry, Technical University of Munich, Lichtenbergstrasse 4, Garching 85748, Germany; § School of Chemistry & Biochemistry, Georgia Institute of Technology, Atlanta, Georgia 30332, United States

**Keywords:** ball mill, mechanical grinding, radical, solid-state chemistry, polyolefin upcycling

## Abstract

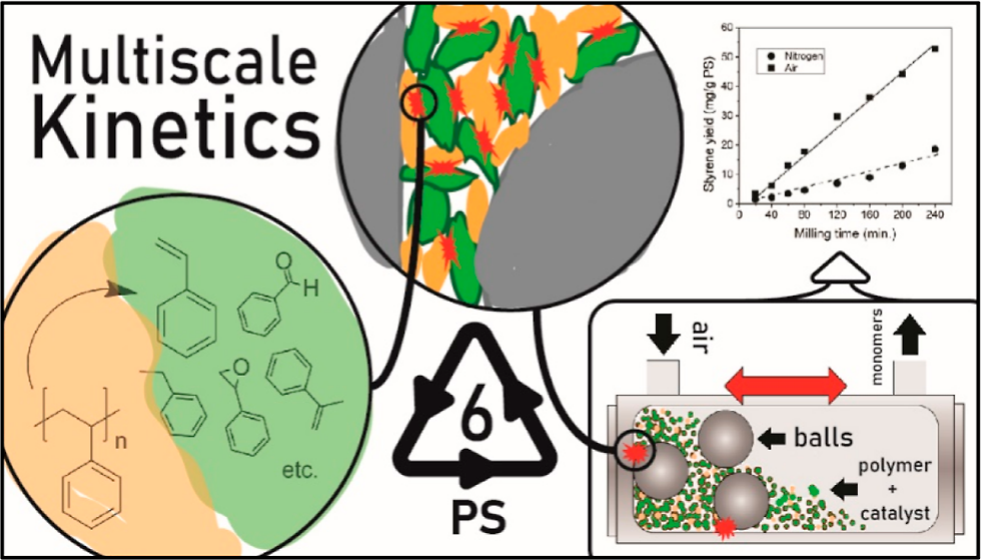

Synthetic polyolefinic plastics comprise one of the largest shares of global plastic waste, which is being targeted for chemical recycling by depolymerization to monomers and small molecules. One promising method of chemical recycling is solid-state depolymerization under ambient conditions in a ball-mill reactor. In this paper, we elucidate kinetic phenomena in the mechanochemical depolymerization of poly(styrene). Styrene is produced in this process at a constant rate and selectivity alongside minor products, including oxygenates like benzaldehyde, via mechanisms analogous to those involved in thermal and oxidative pyrolysis. Continuous monomer removal during reactor operation is critical for avoiding repolymerization, and promoting effects are exhibited by iron surfaces and molecular oxygen. Kinetic independence between depolymerization and molecular weight reduction was observed, despite both processes originating from the same driving force of mechanochemical collisions. Phenomena across multiple length scales are shown to be responsible for differences in reactivity due to differences in grinding parameters and reactant composition.

## Abstract 2

Reaction kinetics involved at multiple length scales in the mechanochemical depolymerization of poly(styrene) are discussed.

## 1 Introduction

Plastics are ubiquitous commodity materials of our age, and plastic waste is a ubiquitous pollutant for our environment. Between 1950 and 2015, around 7800 Mt of plastics were manufactured,^1^ over 80% of which were hydrocarbon polymers collectively termed polyethylenics or polyolefins. Three of them—high and low density poly(ethylene) (HD and LDPE), poly(propylene) (PP), and poly(styrene) (PS)—account for 50% of plastic waste generated annually.^2^ These polyolefins are prevalent in short lifespan material applications such as disposable bags, food containers, and single-use packaging.^3^ Currently, end-of-life processing methods for polyolefin waste are mainly landfilling and incineration, which cause severe environmental harm due to the formation of microplastics and the production of the powerful greenhouse gas carbon dioxide, respectively.^4^ Mechanical recycling via polymer melt extrusion processes about 12% of waste plastics back into commercial products, but this method contends with complications in material behavior due to the presence of chemical additives in commercial polymers, and molecular weight degradation during extrusion undermines the mechanical integrity of the recycled materials, decreasing their economic value.^3,5^

Alternatively, chemical recycling via depolymerization followed by purification of small-molecule products is rapidly gaining attention as the preferred solution for plastic waste processing.^2,3,6^ Depolymerization entails using a chemical reactor to convert waste plastics to small molecules, such as the monomer(s) used to synthesize the plastic in the first place or other primarily liquid or gaseous products of value.^7^ This approach enables a lifecycle for commodity plastics compatible with the circular economy vision espoused by world leaders in science and technology.^3,7^ Research for the depolymerization of polyolefins is mainly focused on catalytic or thermal pyrolysis of the polymer melt.^8^ The pyrolysis route is advantageous in that it is usually an adaptation of petroleum refining technology; thus, its implementation can utilize existing industrial infrastructure and reactor designs.^4,9^ Unfortunately, currently even the best-performing catalytic systems for polyolefin depolymerization require temperatures in excess of 300 °C because polymers remain in a solid state up to hundreds of degrees above ambient conditions, whereas the pyrolysis reactor requires feedstock to be a homogeneous fluid.^10^ Even at elevated temperatures, polyolefin melts are viscous fluids highly susceptible to mass transport limitations when interacting with the pyrolysis catalyst.^11^

A novel alternative to circumventing these issues is to depolymerize polyolefins in the solid state using mechanochemistry, where impact or friction forces between the surfaces of solid reactants provide the driving force for chemical reactions.^12,13^ In recent decades, there has been a proliferation of mechanochemical research in organic synthesis,^14,15^ production of nanoparticles^16^ and solid catalysts,^17,18^ synthesis of metal–organic frameworks^19^ and nanocomposites,^20^ and biomass treatment.^21,22^ Verified mechanochemical reactions involving gaseous reactants and products^23^ include the oxidation of carbon monoxide,^24,25^ water splitting,^26,27^ and ammonia synthesis.^28^ The common thread among all these applications is the use of nominally ambient conditions and a lack of requirement for solvents.

On a lab scale, conditions favorable to mechanochemical reactions are generated in a ball mill, which is a vessel loaded with grinding balls and reactants—usually solid powders. Solid or liquid catalysts can also be added. The vessel is agitated in a rapid periodic motion, which causes impact of the loose grinding balls on the powders.^29^ Common mill types are the attritor, the planetary mill, and the vibratory or shaker mill,^30^ and mills have been the subject of extensive scalability research for industrial applications.^31−33^ Recently, the polyester poly(ethylene terephthalate) (PET) was demonstrated to undergo complete depolymerization when processed in a vibratory mill with sodium hydroxide.^34,35^ By contrast, the lack of labile functional groups on the backbone makes the conversion of polyolefins more challenging. Nevertheless, the breakage of the carbon–carbon bonds of polyolefins through ball milling has been the subject of study since the 1950–60s.^36^ In situ electron spin resonance (ESR) spectroscopy was used to establish that during milling, these polymers break via homolytic scission to form free radical chain ends (so-called mechanoradicals).^37^ Although it is demonstrably feasible to break polyolefin bonds by ball milling,^38^ to the best of our knowledge, only in 2021, ball milling was used intentionally to depolymerize a polyolefin, when Balema et al.^39^ detected styrene in low yield by milling PS. Ball-mill depolymerization of the structurally related poly(α-methylstyrene) (PMS)^40^ as well as oxidative cracking of poly(ethylene) inside a ball mill^41^ have also been reported.

In this work, we study the kinetics of PS depolymerization to styrene in a vibratory ball mill, alongside notable byproducts like benzaldehyde. By quantifying the yield of monomers and molecular weight reduction of the residual polymer as influenced by reactor conditions and catalysts, we identify and systematically classify kinetic phenomena attributable to molecular- and reactor-scale mechanisms as well as intermediate particle-scale effects unique to ball-mill reactors.

## 2 Experimental Section

### 2.1 Materials

Two varieties of PS pellets with average MW = 35,000 g/mol (PS50) and 192,000 g/mol (PS90), methanol (≥99.9%), decane (≥99%), chloroform-*d* (99.8 atom %), styrene (≥99%), chromium (<45 μm powder, ≥99%), iron (<10 μm powder, ≥99.9%), nickel (<50 μm powder, 99.7%), boric acid (≥99.5%), iron(III) oxide (<5 μm powder, ≥96%), and barium titanate (<3 μm powder, 99%) were purchased from Sigma-Aldrich. Ultra zero grade air and ultrahigh purity grade N_2_ and O_2_ gases were purchased from Airgas. All chemicals were used as received without further purification.

### 2.2 Mechanochemical Reactions

Experiments were conducted in a Retsch MM400 shaker mill using custom-machined 316-grade stainless steel vessels and grinding balls. The reactor vessels had a pill-shaped internal volume of 25 mL and Swagelok-fitted openings on the cylindrical face for the optional gas flow. Grinding balls with masses ranging from 1.38 to 63.1 g were used in experiments, with 4.04 g (10 mm diameter) balls being the usual choice. PS pellets were premilled into a coarse powder prior to depolymerization reactions using the following specifications: 10 g of PS pellets and two 20 mm diameter stainless steel balls were loaded into a 50 mL Retsch steel reactor and milled at 30 Hz for 30 s (PS50) or 4 min (PS90). The effect of premilling on the molecular properties of PS90 was found to be insignificant (see Figure S1f). In a typical experiment, the 25 mL reactor vessel was charged with 1 or 8 grinding balls of uniform size, 1 g of PS and optionally less than 1 g (usually 0.1 g) of a chemical catalyst, and then mounted onto the mill. The reactor was connected to 1/8th in. teflon tubing using Swagelok unions for gas flow experiments, with flow rate controlled using a mass flow controller upstream from the inlet line. Downstream from the outlet, a gas dispersion tube was used to bubble effluent gas from the reactor into a methanol (MeOH) solvent trap containing 1 mg of decane as an internal standard. For NMR spectroscopy samples, the gas trap solvent was chloroform (CDCl_3_) instead of MeOH, and no internal standard was used. The reactor was purged by N_2_ gas for 5 min prior to the commencement of milling. Milling was performed continuously without interruption for a specified reaction time and set of conditions. At the conclusion of an experiment, 1 mg of decane and 6–7 mL of MeOH were added into the reactor, and the contents were shaken on the mill at 10 Hz for 1 min to generate a suspension of solid polymer particles in liquid. Liquid samples were prepared by passing 1 mL of the suspension from the reactor or the MeOH gas trap solution through a 0.2 μm PTFE syringe filter into a chromatography vial. The solid residue was collected from the suspension liquid via suction filtration and dried overnight in a fume hood.

### 2.3 Characterization of Materials and Reaction Products

Gas chromatography (GC): an analysis of the liquid samples was performed on a Varian-Bruker 450-GC instrument equipped with a Supelco SPB-1 fused silica capillary column, a Polyarc quantitative carbon analyzer manufactured by Activated Research Company, and a flame-ionization detector (FID). The carrier gas was helium at a rate of 2 mL/min. Samples taken from experiments were analyzed within 6 h from the end of the reaction. The quantitative carbon analyzer allowed for quantification of the amounts of reaction products in the liquid fraction based on the relative intensity of FID signals and the amount of internal standard present in the sample. FID signal positions of detected compounds were calibrated using samples of the corresponding pure compound. The yield (product mass per unit mass of PS) of each product compound *i* was calculated using its peak integration area *I*_*i*_, molecular weight MW_*i*_, and carbon number *N*_*i*_ and the same quantities (*I*_dec_, MW_dec_ = 142.3 g/mol, *N*_dec_ = 10) for decane, normalized by the initial mass of PS *m*_PS,0_ in the reactor, according to the following formula
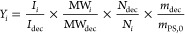
1

Instrumental specifications for the characterization of solid samples by gel permeation chromatography (GPC), liquid fraction samples by gas chromatography–mass spectrometry (GC–MS), and assorted samples by nuclear magnetic resonance (NMR) spectroscopy, attenuated total reflectance Fourier-transform infrared (ATR-FTIR) spectroscopy, and dynamic light scattering (DLS) are provided in the Supporting Information, Sections B–F, respectively, for these techniques.

## 3 Results

### 3.1 Reaction Scheme

**Figure 1:**
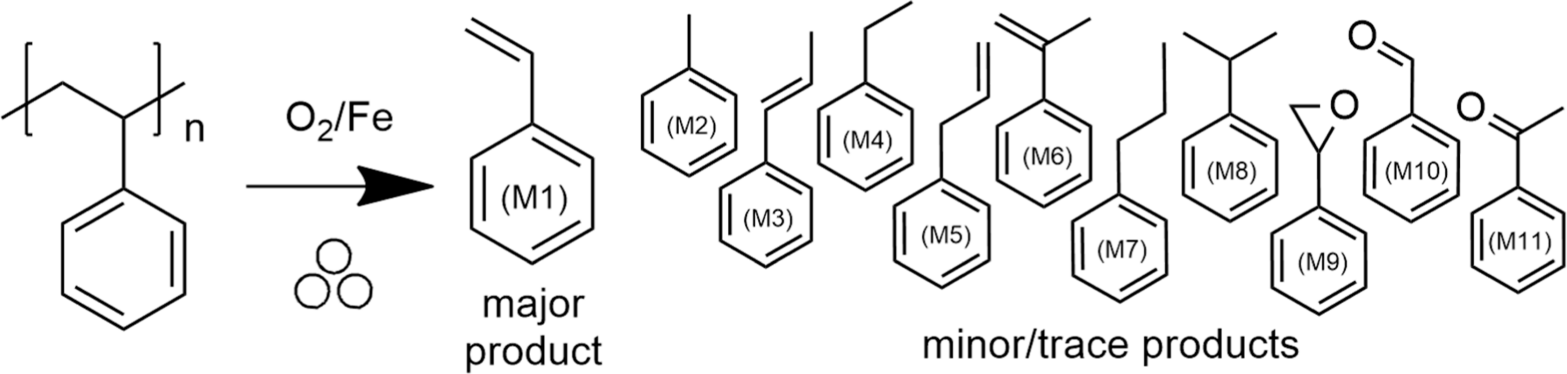
Products of the mechanochemical depolymerization of PS.

Ball milling of PS generated a solid polymer residue and small molecule products in CDCl_3_ or MeOH solutions. Chemical structure analysis of these respective solutions by NMR spectroscopy or GC–MS revealed styrene (M1) as the primary product (see Supporting Information, Sections S.C., Table S2 and S.D., and Figures S3C and S4C). Aromatic hydrocarbons toluene (M2), propenylbenzene (M3), ethylbenzene (M4), allylbenzene (M5), α-methylstyrene (M6), and *n*-propylbenzene (M7) were also detected in minor or trace quantities. Minor products with functional groups were the oxygenates styrene oxide (M9), benzaldehyde (M10), and acetophenone (M11). Henceforth, these depolymerization products shall be termed “monomers”. The reaction scheme is depicted in Figure 1, with the conventional “three balls” symbol indicating mechanochemical conditions.^42^

### 3.2 Time Progression of Monomers

**Figure 2:**
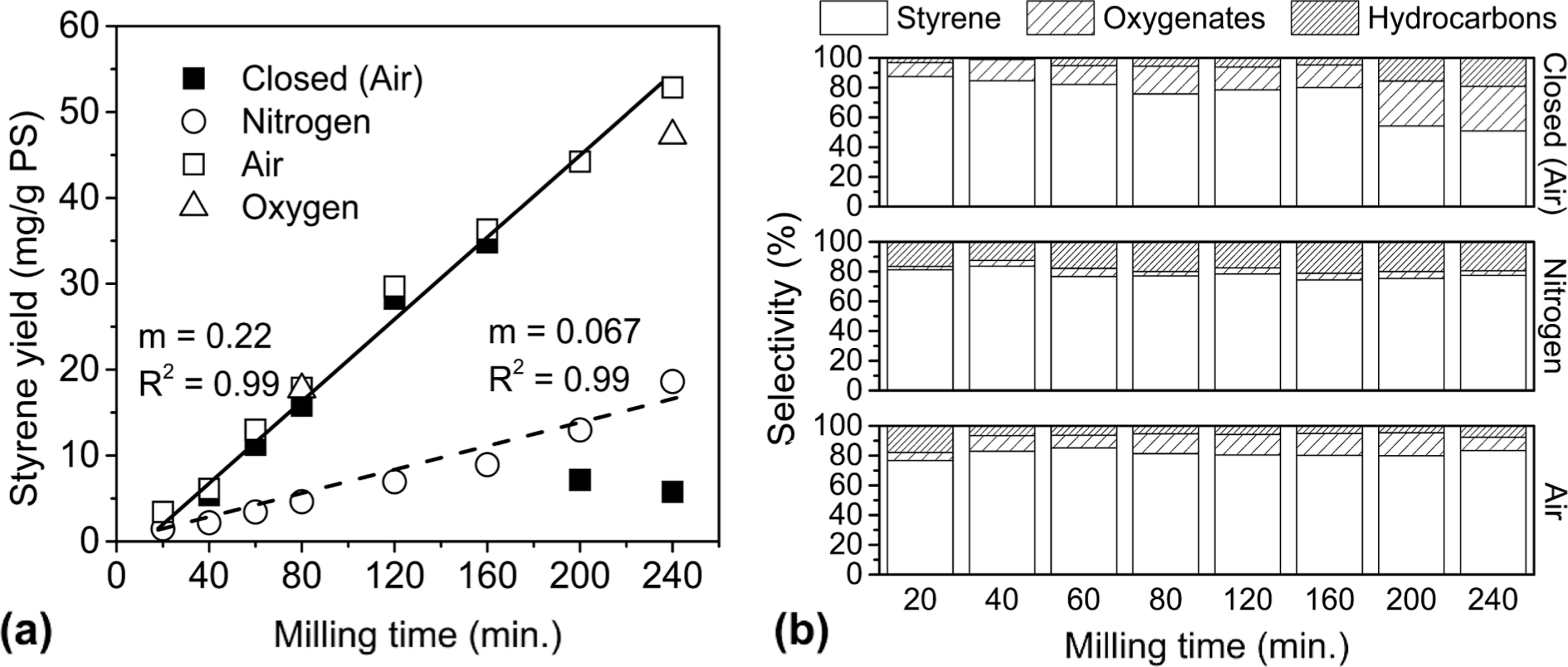
Depolymerization of PS90 using a 25 mL stainless steel flow reactor, eight 10 mm stainless steel balls, and 1 g of PS90, milled at 30 Hz. (a) Yield vs time for PS90 cl., op.a, op.n, and op.o with 20 sccm gas flow. Lines of best fit are drawn for cl. + op.a data (excluding two cl. data points at longest time) and op.n data. (b) Product selectivities for cl., op.n, and op.a conditions.

For PS90, the styrene yields and product selectivities with milling time under various gas phases are depicted in Figure 2. Two types of reactors were used: closed conditions refer to milling in sealed reactors containing ambient air as the initial gas phase. Open conditions were achieved by flowing either air, pure N_2_ or pure O_2_, through the reactor during milling. As a useful shorthand, closed conditions will be denoted by “cl.” when referred to in figure captions and certain paragraphs, and open conditions will be denoted by “op.n”, “op.a”, and “op.o” for milling under nitrogen, air, and oxygen flow, respectively.

Initially, the styrene yield increased linearly with milling time, indicative of a constant monomer production rate. For a closed vessel (Figure 2a, black squares), this linear relationship persisted up to around 160 min, when the amount of accumulated styrene in the reactor was 35 mg/g PS. Between 160 and 200 min, however, the amount of styrene in the reactor collapsed to under 10 mg/g PS. No such collapse was observed in any experiment that did not exceed 30 mg/g PS in styrene yield. Moreover, when 35 mg of styrene was mixed with PS prior to milling, the final styrene accumulation after 20, 40, and 80 min of milling in a closed vessel was indeed measured to be less than the initial amount (see Table S1, entries marked “PS90 + M1 cl.”).

By contrast, the styrene yield achieved in an open vessel with air flow (Figure 2a, hollow squares) was measured based on the sum of the styrene accumulated in the reactor and in the effluent gas trap, and a constant styrene production rate was preserved throughout the entire period of 240 min. Initially, the majority of styrene was collected from the reactor, but by 120 min, styrene accumulation inside the reactor reached a plateau, and increasing quantities were accumulated in the gas trap (Table S1).

Like styrene, oxygenates (mostly benzaldehyde and styrene oxide, the next two most abundant products) exhibited constant rates of formation. Unlike with styrene, in the closed reactor, the quantities of these monomers did not collapse between 160 and 200 min, but their accumulation tapered off after styrene accumulation had collapsed (see Table S1). In air flow, constant rates for byproducts were maintained, akin to styrene, even though fluctuations in measured yields were higher. Compared to styrene, oxygenates exhibited greater accumulation in the reactor than in the effluent stream, consistent with the relative volatilities of these products, with styrene being the most volatile and hence accumulating more in the effluent trap, whereas progressively less volatile oxygenates such as benzaldehyde and styrene oxide stayed in the reactor in greater proportions. However, notwithstanding the changing product composition inside the reactor, when the reactor and effluent portions were summed, the selectivities of these monomers all remained approximately constant with time, with around 80% styrene, 15% oxygenates, and hydrocarbons for balance. Approximately constant selectivity was also observed across closed reactor experiments prior to the styrene collapse, as shown in Figure 2b.

The presence of oxygenates was indicative of the presence of O_2_ from the air, which plays a role in the depolymerization mechanism. To confirm the significance of O_2_, experiments under N_2_ flow (Figure 2a, hollow circles) were conducted to minimize the presence of O_2_. The result was a reduction of the styrene production rate by a factor of 3.3, which still remained constant with time. Despite milling in a nominally oxygen-free environment, some oxygenates were still detected in the products, but at a greatly reduced selectivity of only 1–3%. The oxygen source may be surface-adsorbed species on the metal grinding surfaces or oxygenated additives in PS.^43^ The ATR-FTIR spectrum of PS90 (Figure S5) does indeed exhibit slight bands around 1350 and 1150 cm^–1^ suggestive of secondary and tertiary alcohol groups. The selectivity of styrene was also reduced to around 76% at the expense of increased amounts of other hydrocarbons (Figure 2b). Further increasing oxygen content with O_2_ flow (Figure 2a, hollow triangles) produced styrene yields identical to air flow at 80 min and slightly lower at 240 min.

### 3.3 Characterization of Polymer Residue

**Figure 3:**
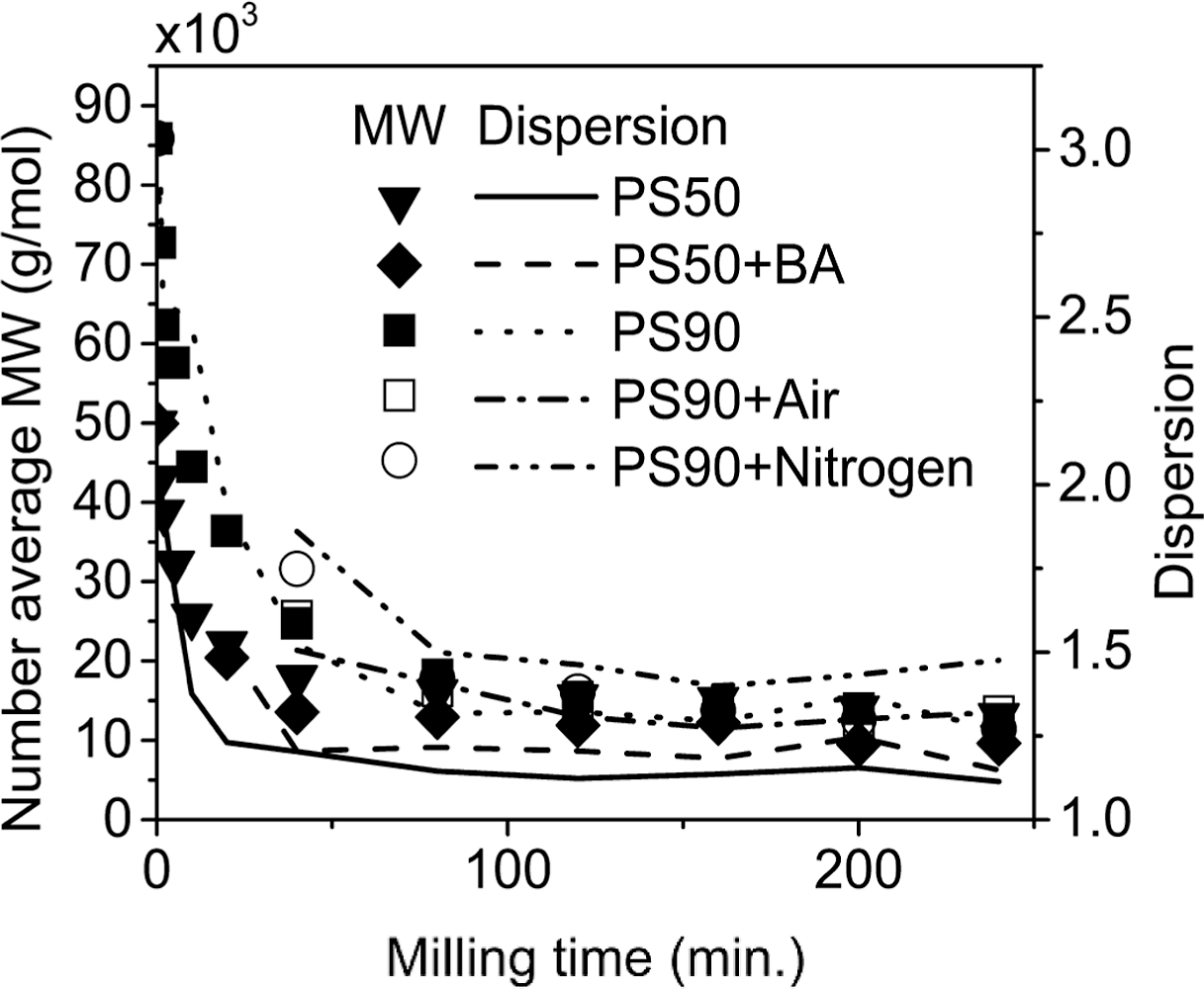
*M*_N_ and PDI vs time using a 25 mL stainless steel reactor, eight 10 mm stainless steel balls, milled at 30 Hz, 1 g PS90 was milled under cl., op.a., and op.n conditions, PS50 was milled under cl. conditions with and without boric acid (BA). MWs calculated from DLS detector.

The number-average molecular weights (*M*_N_) of the solid residues from PS90 as determined by GPC are depicted in Figure 3 (black and hollow squares and hollow circles). A rapid decrease in MW was observed within the first hour of milling, as expected from successive mid-chain scissions. Subsequently, MW reduction slowed considerably at around *M*_N_ = 10,000 g/mol. This trend was observed irrespective of milling in a closed reactor under air or N_2_ flow. For milling times longer than 120 min, variation in MW in experiments involving different gas phases was generally within ±1000 g/mol and could be attributed to the margin of error in the measurement. In more detailed MW distribution curves (Section S.B., Supporting Information), a consistent but small shift of the entire distribution to lower MW was perceptible with an increase in milling time. No obvious effect on MW was observed concurrent with the collapse in styrene accumulation that occurred between 160 and 200 min under closed conditions.

**Figure 4:**
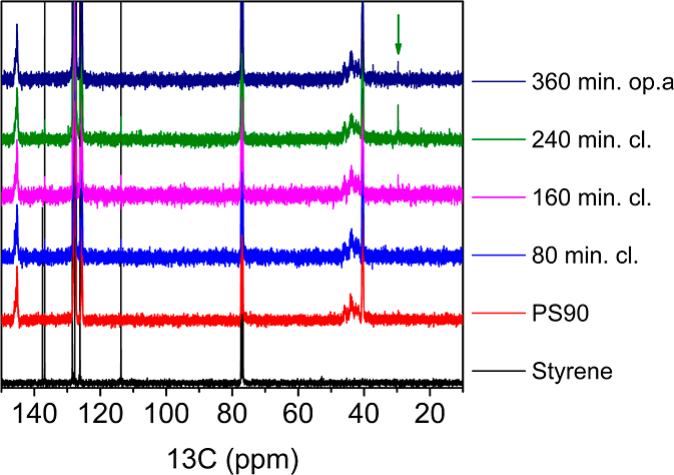
Stacked ^13^C NMR spectra of monomer styrene, unmilled PS90, and PS90 milled under the following conditions: 80 min cl., 160 min cl., 240 min cl., and 360 min op.a. A green arrow at 30 ppm indicates the location of an unambiguous new signal in samples milled for a long time.

^13^C NMR (Figure 4) spectra were taken for unmilled PS90, and residues from milling were measured in the closed vessel for 80, 160, and 240 min, in nitrogen flow for 240 min, and in air flow for 240 and 360 min. The samples of PS90 taken after long milling times exhibited a yellow tint when dissolved in CDCl_3_, whereas the solution of the unmilled polymer was colorless. The spectra of milled samples appeared identical to that of the unmilled polymer except for the gradual appearance of an unambiguous signal at 30 ppm (indicated by a green arrow, top right of Figure 4), assignable to an aliphatic carbon, which increased in height with an increasing milling time. With all polymer spectra scaled to the height of the 40 ppm band of PS, the peak at 30 ppm under closed conditions was not yet distinguishable from the baseline after 80 min but became visible by 160 min and more than doubled in height by 240 min, coinciding with the collapse in styrene concentration in between these time points. By contrast, after milling under air flow for 360 min, this peak was only comparable in magnitude to 160 min under closed conditions. The spectrum of the samples milled for 240 min op.a and 240 min op.n were practically identical to that of the unmilled polymer (see Figure S3I,J), indicating that the emerging carbon moiety corresponding to the 30 ppm peak required very long milling times to become visible under open milling conditions and irrespective of the type of gas flowed, whereas it grew much faster under closed milling conditions. Additional signals not present in unmilled polymer were observed in the aromatic region (110–140 ppm); however, when overlaid against the spectrum for monomer styrene, these signals formed an exact match against the most prominent signals of styrene (left side in Figure 4), indicating the presence of residual styrene monomers in all the milled polymer samples.

### 3.4 Effect of Catalyst and Catalyst Proportions

**Figure 5:**
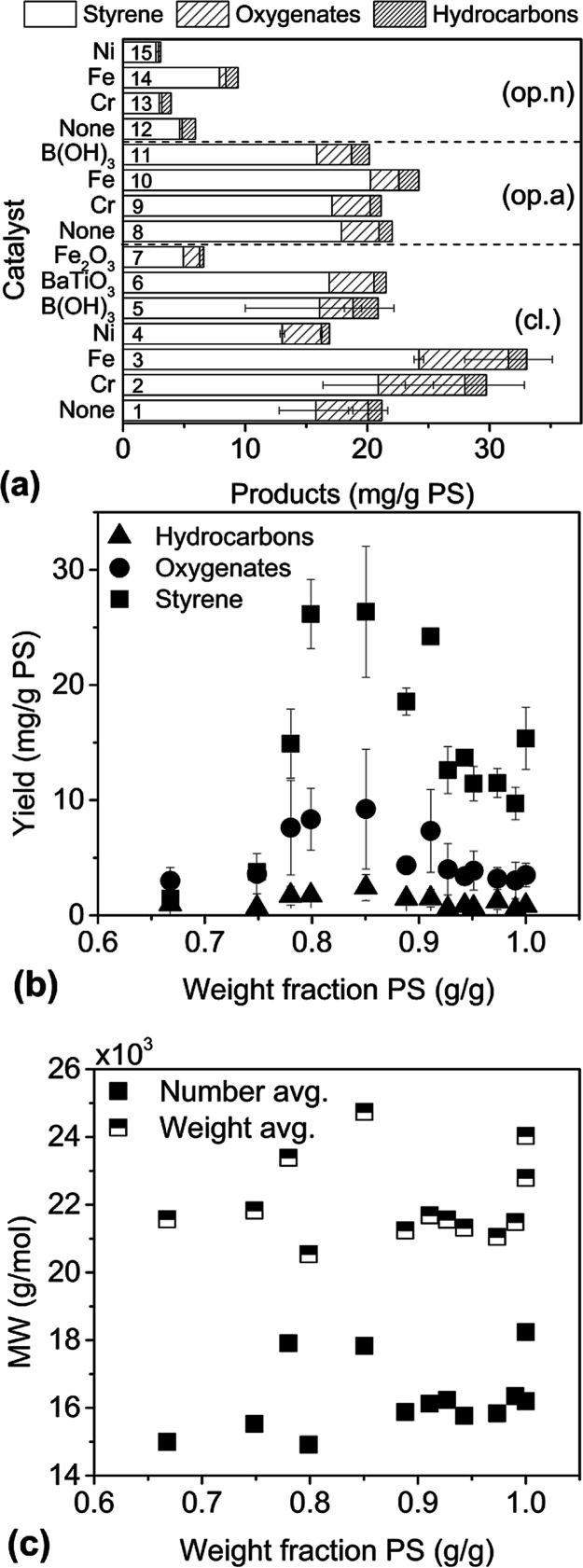
Depolymerization of PS90 using a 25 mL stainless steel reactor, eight 10 mm stainless steel balls, and 1 g of PS90 milled cl. at 30 Hz for 80 min; (a) includes 0.1 g of additive, experiments are grouped by cl., op.a, or op.n depending on the reaction conditions, (b) styrene yield for various proportions of Fe powder at 80 min cl., and (c) MW vs proportions of Fe powder.

Using stainless steel grinding equipment, styrene was produced even under a N_2_ atmosphere. Therefore, some constituent(s) of stainless steel may play a chemical role in depolymerization through surface interactions with the PS particles. Thus, by adding a chemical catalyst powder to the reactor, styrene production may be enhanced during milling due to the increased surface interactions between PS and the catalyst particles. Several candidate materials were milled with PS90 to gauge their catalytic ability, including the three main metal components of 316-grade stainless steel: Fe, Cr, and Ni metals, as well as several inorganic materials that have appeared in the mechanochemistry or depolymerization literature in other contexts: piezoelectric barium titanate BaTiO_3_,^44^ solid acid boric acid (BA),^45^ and iron(III) oxide Fe_2_O_3_.^41^ Holding other parameters constant, monomer yields for PS90 milled with these additives are shown in Figure 5a.

The results for closed reactor milling showed that Cr and Fe are the catalytically active components in stainless steel–albeit neither are selective toward specific products since both styrene and byproduct yields increased compared to experiments in their absence (compare entries 1–3). On the other hand, Ni slightly reduced the yields (entry 4). Enhancement in the styrene yield using either Cr or Fe was less significant under air flow (entries 9 and 10), which could indicate that the catalytic activity is lost when these metals are oxidized. BA under closed or air flow conditions (entries 5 and 11) and BaTiO_3_ (entry 6) had no apparent effect on the yields, while Fe_2_O_3_ (entry 7) significantly reduced yield. Any intrinsic catalytic effects of additives should be revealed under N_2_ flow when the synergistic effects of oxygen are eliminated. It was found that Fe (entry 14) still enhanced the styrene yield relative to control (entry 12), but neither Cr nor Ni (entries 13 and 15) exhibited such an enhancement, and their presence in fact suppressed monomer production.

As a consequence of the particulate nature of reactants in mechanochemistry, ball mill depolymerization of PS must be regarded as a two-phase solid-state reaction, with the second phase being the metal (or other material) surfaces that can serve as catalysts for monomer production.^42^ In such two-phase systems, only heterogeneous phase contacts will lead to reactions, which means physical contact between two PS particle surfaces is unlikely to contribute significantly to depolymerization. Therefore, by adjusting the amount of each powder present in the reactor, it is possible to optimize for desirable PS-catalyst surface contacts (i.e., particle mixing) that lead to depolymerization. This was done with PS90 and Fe powder under closed conditions, and the resultant styrene yields are plotted in Figure 5b. These results reveal that an optimal ratio of Fe to PS exists between 80 and 85% PS by the total weight of powder. Increasing Fe content further than 20% by weight led to a drastic reduction in styrene yield, and decreasing Fe resulted in a roughly linear reduction in yield down to the intrinsic value when PS was milled by itself, albeit with a large spread in the data points.

The MWs of all PS samples milled with Fe are shown in Figure 5c, and the results reveal that for Fe used within 10% by weight, *M*_N_ only decreased slightly by 2000 g/mol relative to milling PS by itself, while for higher percentages of Fe, greater spread in measured MW was observed.

### 3.5 Effect of Mechanical Energy Supply

At the reactor level, mechanochemical kinetics in a vibratory ball mill are controlled by the mechanical energy that is transferred during the collision of grinding surfaces.^30,46,47^ This was quantitatively demonstrated by Tricker et al. in the depolymerization of PET with NaOH.^35^ From elementary mechanics, the instantaneous kinetic energy of a grinding ball traveling inside the vibratory mill in between collisions is given by eq 2
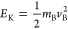
2where *m*_B_ is the mass of the ball and *v*_B_ is its speed. When a ball collides with another grinding body with kinetic energy *E*_K_, a part of that energy can be used to drive chemical reactions in the material caught in between.^46^ Ball motion expressed through *v*_B_ is imparted by the motion of the reactor that is governed by the mill frequency *f*_M_. Except for the case of a single-ball reactor, the motion of grinding balls is generally random and chaotic during reactor operation; therefore, analytical expressions for *v*_B_ as a function of *f*_M_ are difficult to obtain for ball mills with multiple balls.^48^ Nonetheless, *v*_B_ (and, by extension, *E*_K_) generally increases with *f*_M_
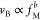
3where *b* > 0 is an empirical constant. Therefore, *E*_K_ can be tuned most directly by changing *f*_M_ and by changing ball mass *m*_B_.^49^ Thus, the influence of the mechanical energy supply on PS depolymerization kinetics was investigated by varying *f*_M_ and using balls of different sizes to adjust *m*_B_. In these experiments, the only products that formed consistently at measurable quantities were styrene and benzaldehyde so only these products are reported in the subsequent discussion and figures.

**Figure 6:**
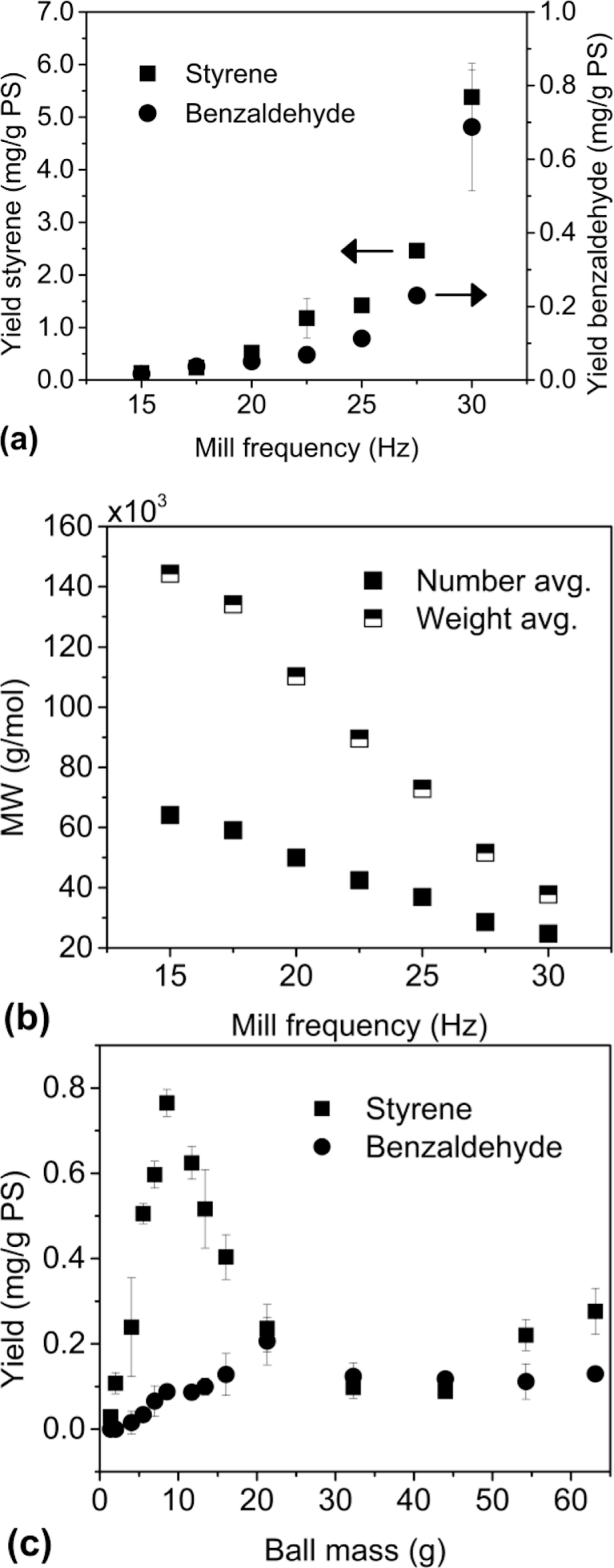
Depolymerization of PS90 using a 25 mL stainless steel reactor, eight 10 mm stainless steel balls, and 1 g of PS90 milled cl. for 40 min; (a) yield of styrene and benzaldehyde (two most abundant products) vs milling frequency using eight 10 mm diameter balls, (b) corresponding MWs for experiments in (a), and (c) yield of styrene and benzaldehyde vs ball mass, milling conducted at 30 Hz using 1 ball.

The relationship between *f*_M_ and the styrene yield (Figure 6a) was nonlinear, but an increasing yield was unambiguously observed with increasing milling frequencies up to the maximum frequency of the equipment of 30 Hz. As with styrene, the most abundant byproduct benzaldehyde was also produced in increasing quantities with higher *f*_M_. The shape of the yield versus frequency curves for both products appeared to be similar. Since increased energy supply increases the frequency of chemical events (in this case, chain scissions), the MW decreased as expected with increasing frequency (Figure 6b). Contrasting Figure 6b against Figure 5c, it is clear that the reactor parameter of the energy supply has a far greater influence on MW progression than the presence of chemical additives, even though both significantly affected monomer yields.

For experiments varying *m*_B_, one stainless steel ball of different sizes was used, and the styrene yield (Figure 6c) increased as *m*_B_ increased from 1.38 to 8.56 g, but then decreased from there to the 32.25 g ball. The subsequent increase in yield beyond 40 g might also be a statistically significant result. The reactors used in these experiments have a cross-section of only 1 in., so a 1 in. diameter (63.1 g) ball represents the largest sized ball that could fit into the reactor. Based on these considerations, it can be stated conclusively that styrene yield was optimal for *m*_B_ centered in the vicinity of 8.56 g, with decreasing trends in yield going in either direction. As with styrene, benzaldehyde also appeared to exhibit a maximum yield, but only at a higher *m*_B_ of around 21.3 g instead of 8.56 g for styrene.

### 3.6 Conversion of Bimodal PS

**Figure 7:**
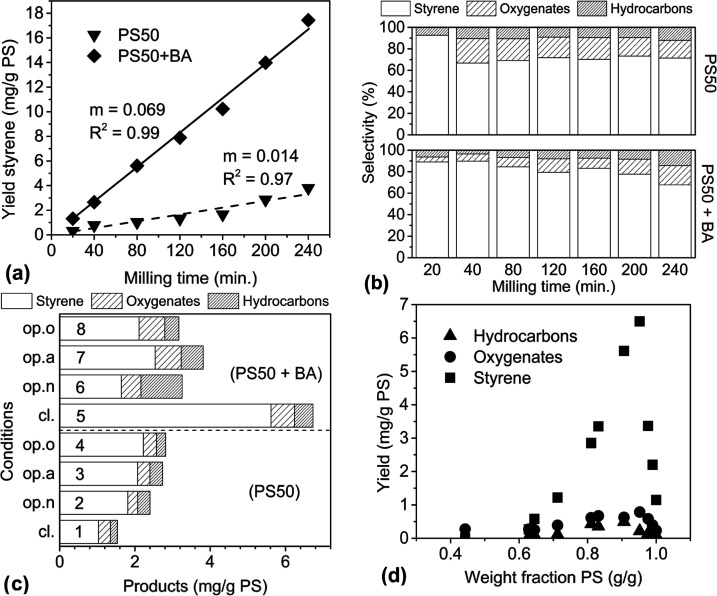
Depolymerization of PS50 using a 25 mL stainless steel reactor, eight 10 mm stainless steel balls, and 1 g PS50 (+0.1 g BA), milled at 30 Hz; (a) styrene yield with time under cl. conditions, (b) corresponding selectivity for PS50 by itself and for PS50 + BA, (c) styrene yield for closed and open (20 sccm gas flow) conditions with and without BA, and (d) variation in proportion of PS50 to BA under closed conditions.

To provide comparison to the depolymerization kinetics of unimodal PS90, a bimodal PS50 containing fractions with MWs of 1000 and 50,000 g/mol with an approximate ratio of 55:45 (as determined by GPC) was milled in a closed vessel using the same parameters as for PS90. The time progression of the styrene yield and monomer selectivity are shown in Figure 7a,b. Although lower quantities of monomers were produced from PS50 compared to PS90, a constant monomer production rate was also observed for this sample, with styrene selectivity at around 70%. An increased yield of α-methylstyrene in the hydrocarbons made up the difference (see Table S1).

While BA did not influence the monomer yield from PS90, the presence of 10 wt % of BA increased the styrene yield from PS50 in a closed vessel by a factor of 5 compared to pure PS50. As in other cases, a constant rate of monomer production was observed. However, the selectivity for styrene gradually decreased with time in the presence of BA, despite the preservation of the constant styrene production rate, with increasing quantities of oxygen detected at long milling times.

The MW progression (Figure 3, inverted triangles and diamonds) for the 50,000 g/mole fraction of PS50 exhibited the same behavior as that of PS90. Specifically, the MW decreased rapidly within the first 2 h of milling and then decreased slowly once it approached 10,000 g/mol. The presence of BA led to a slightly lower MW at each time point. On the other hand, the oligomeric fraction of PS50 (around 1000 g/mol) did not exhibit any significant change in MW as measured by GPC for any duration of milling, and was not included in the plot (see Figure S1a–e). PS90 and PS50 both started out with PDI greater than 2, milling for 4 h narrowed the PDI to 1.3 for PS90 and 1.2 for the high MW fraction of PS50.

Flowing gases uniformly improved the styrene yield from PS50 (Figure 7c, entries 1–4). A slight increase in styrene yield was observed on going from N_2_ to air to an O_2_ flow. By contrast, although the addition of BA boosted styrene yield under closed conditions, a less impressive improvement was seen under any gas flow (Figure 7c, entries 6–8). In fact, under gas flow, BA appeared to lose all effectiveness at increasing the styrene yield, irrespective of gas type. However, the production of byproducts was more pronounced compared with closed conditions.

Finally, the effect of adjusting the proportions of PS50 to BA was investigated (Figure 7d). The optimal yield was obtained at 0.96 weight fraction of PS50 (or 4% BA by weight). Increasing the amount of BA above this maximum resulted in a more steady decline in yield relative to the rapid drop observed for PS90 with Fe. This in turn meant a sharp boost in yield in going from PS50 milled alone to just a small amount of BA.

## 4 Discussion

### 4.1 Reactor-Scale Kinetics

At a reactor-scale, mechanochemical processes are commonly described based on the material properties of reactants (such as molecular weight, characteristic mechanical properties, etc.) and reactor-averaged density or frequency variables (such as monomer concentration and macroscopic temperature).^42^ In the present study, the most pervasive result across all sets of experiments conducted under fixed reactor conditions (Figures 2a and 7a) was the constant rate of monomer formation with time. The collapse in styrene concentration between 160 and 200 min of milling inside the closed reactor can be explained by reaching a threshold concentration for runaway macroscopic repolymerization. For a polyolefin, the thermodynamic equilibrium between polymerization and depolymerization is characterized by the ceiling temperature when the polymer is in equilibrium with its monomer. Depolymerization is favored over polymerization above this temperature. Since the macroscopic temperature inside the reactor is nominally ambient and therefore well below the ceiling temperature range of PS (310–395 °C^50,51^), polymerization of monomers is thermodynamically favored over depolymerization. Monomer loss through repolymerization is insignificant when the concentration of styrene is kept low by its removal from the reactor in a gas stream. However, in the closed reactor, styrene concentration increases without restriction, and the conditions of repolymerization are easily satisfied (Section 4.3). Depolymerization without impediments was achieved by implementing an open reactor configuration with a purge gas stream to remove volatile products, resulting in the preservation of the constant rate of styrene formation.

In contrast to the constant rate of monomer production with milling time, the MW reduction of PS converged at around 10,000 g/mol regardless of the starting MWs and different processing conditions (Figure 3). In fact, this was also the lower limit in MW obtained by Staudinger in 1934 upon subjecting different types of PS to mechanical abrasion.^52^ The reason for this apparent lower limit in MW is the existence of a critical molecule size in solid polymer particles, above which rupturing a chemical bond via the applied mechanical forces becomes more energetically favorable than disrupting the physical van der Waals interactions of the halves of the polymeric molecules with their environment.^37^ Indeed, in the case of the bimodal PS50 material, only the high MW fraction of PS50 underwent MW reduction (Figure S1a–e), although the oligomeric fraction was not chemically inert in the presence of BA (Section 4.3).

**Figure 8:**
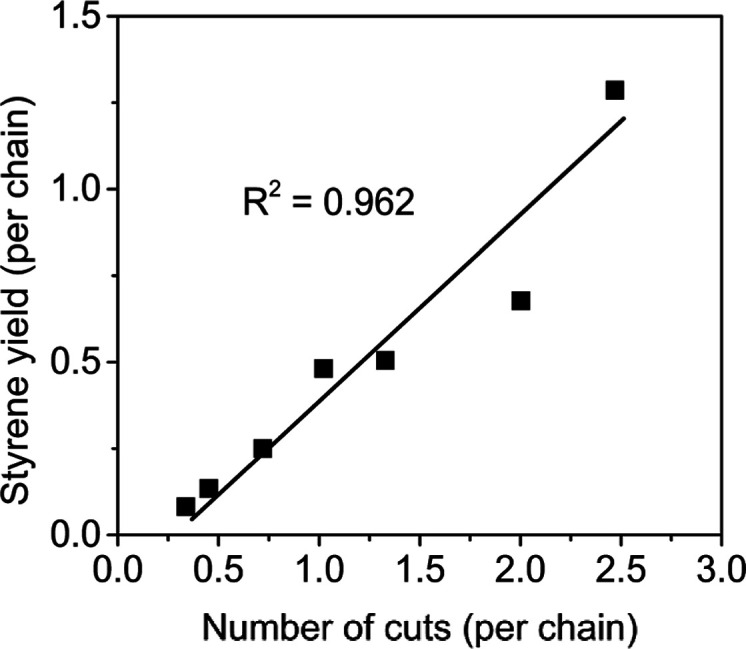
Styrene yield (*y* axis) as a function of MW reduction (*x* axis) for milling at different frequencies, with both quantities normalized by number of chains counted after milling using number-average MW. Milling time (40 min), number (8) and size (10 mm) of balls, and mass of PS90 (1 g) kept constant across all experiments.

It is expected for MW reduction and monomer production to be qualitatively asynchronous in time; however, they can be correlated for energy supply, a characteristic descriptor of reactor performance-dependent on all the operating parameters, including reactor geometry, number, size, and density of grinding balls, milling frequency, and reaction time.^30,46,53^ In our study, we investigated the influence of the energy supply on kinetics by adjusting the milling frequency *f*_M_. Using the data for styrene production and MW as functions of *f*_M_ in Figure 6a,b, respectively. These experiments were performed for a short milling time (40 min) when both scission and monomer production occurred simultaneously. For each experiment, using the initial and final *M*_N_, we can calculate the number of cuts the average chain had undergone during milling. Meanwhile, by dividing the number of styrene molecules produced in each experiment by the number of chains present at the end of milling in that same experiment, we obtain the average number of styrene monomers yielded per chain. For different *f*_M_, we plot in Figure 8 this pair of values against each other and find that their magnitudes are both on the order of 1, which indicates that MW reduction and monomer production occur at a similar rate during the first 40 min of milling when both quantities are normalized on a per chain basis. Figure 8 reveals a linear correlation, signifying that the energy input—which is tuned by adjusting milling frequency *f*_M_—serves as the driving force for both MW reduction and monomer production. As milling time increases, MW reduction becomes less prevalent (in Figure 8, the positions of the data points along the horizontal axis would remain fixed), but that does not interfere with monomer production (the data points would shift upward along the vertical axis), which continues unabated.

The monomer yields in reactions with different ball masses, *m*_B_ (Figure 6c), reveal another aspect of the kinetics at the reactor level. The data could be divided into three regimes. For balls less than 8.56 g, the yield of either monomer increased approximately linearly with mass, a result that agrees with a previous study on PET depolymerization.^35^ Increasing the ball mass past the “optimal mass” led to a decrease in yield, which stabilizes to a near-constant value by the third regime when the size of the grinding ball grows closer to the cross-section diameter of the reactor, rounded out by a final uptick in yield for the largest possible ball that can fit inside the reactor. The first regime at low ball masses follows the expected behavior from the frequency results, where increasing *E*_K_ by increasing *m*_B_ (instead of *f*_M_) leads to increased monomer production, but the subsequent regimes, where the trend reverses and then stabilizes, require other explanations.

First, the monomer yield with the highest ball mass (largest ball) can be rationalized as a geometric effect. Although the motion of a grinding ball in a vibratory mill is generally random, on average the path is from one end of the cylindrical reactor to the other and back, so randomness in its path of motion decreases as the size of the ball increases until finally the ball fits exactly into the reactor’s cross-section without significant clearance.^49^ At this point, the motion of the ball is exactly synchronous with the motion of the reactor, while simultaneously reactant particles are statistically unlikely to parry the ball’s impacts due to the geometric constraints present. The result of these conditions is that the amount of chemical reaction per grinding surface collision is increased because the number of particles subjected to each collision is maximized.

However, before this regime is attained, there is an even more significant decrease in yield with increasing ball mass, which can be rationalized as being due to changes in the mechanical properties of PS with temperature. In this study, the temperature on the exterior surface of the reactor when milling with eight 4.04 g balls equilibrated to about 40 °C at long milling times, whereas a gradual but steady increase in temperature from the ambient to greater than 50 °C was observed when milling with just one 13.42 g ball for only 1 h. Since the external surface temperature represents only a portion of the total heat generated in the grinding collisions, the interior surfaces of the reactor must be hotter. Furthermore, it has been demonstrated that transient thermal hot spots formed during collisions can reach temperatures much higher than the bulk temperature in a ball mill.^54^ We can therefore deduce that greater heat accumulation occurs in the reactor with increasing mass of the ball used in milling, consistent with results from ball milling studies that have attempted to quantitatively estimate heat generation due to collisions.^55,56^ On the material side, the characteristic property that delineates when a polymer undergoes dramatic changes in its mechanical properties is the glass transition temperature. Well below the glass transition, PS is a hard, brittle solid, whereas approaching and above the transition point, it becomes soft and ductile.^57^ PS has its glass transition temperature range in the neighborhood of 100 °C,^58^ which decreases with decreasing MW.^59,60^ Thus, there is a gradual convergence between the glass transition temperature region of the material and the temperature on the internal surfaces of the reactor.

When pressed together from above and below, hard and brittle particles will generate friction from mechanical contact between their surfaces and rupture into smaller pieces—generating fresh surfaces—if loaded above their ultimate strength.^57^ Meanwhile, soft and ductile particles subjected to the same compression will deform plastically.^61^ In the former case, energy transfer from grinding surfaces to reactant is predominantly located on the surfaces of particles, whereas in the latter case it is into the bulk interior of the reactant material (plastic deformation being a process acting on the material as a whole rather than just on its surfaces). Where liberation of volatile products is involved, mechanochemical reactions of particles constitute a predominantly surface or near-surface transformation;^62,63^ therefore, it follows logically that milling of PS particles in its hard and brittle state, where friction and fracture dominate is more conducive to monomer production than milling in the soft and ductile state, where a greater share of the grinding energy supply is dissipated into the bulk of the material during plastic deformation. With heavier balls creating higher surface temperatures in the reactor that then heat the polymer particles toward a physical state where their mechanical properties no longer favor monomer generation, we can explain the decline in yield with increasing ball mass above the point where energy supply is optimal. Stabilization in monomer yield that occurs in the subsequent, highest ball mass regime can be regarded as a balance between the constructive geometric effect, which makes collisions more efficient by impacting more particles, and the detrimental temperature-induced mechanical softening effect, rendering depolymerization less favorable.

### 4.2 Particle-Scale Kinetics

Unlike ideal chemical reactors, such as the continuous-stirred tank reactor (CSTR) or plug flow reactor (PFR), where the reactant phase is a homogeneous fluid, the ball-mill reactor is a heterogeneous multiphase system, where chemical reactions occur at interfaces between solid particles.^62^ For this reason, chemical reaction events cannot be considered solely on the basis of the probability of collisions of two molecular species to undergo a desired reaction but also the probability of two solid particles containing the desired reactive species on their surfaces when coming into physical contact mechanochemically.^42^ Mechanochemical contact between reactant particles occurs when they are crushed together by a collision of grinding bodies. The number of collisions during the course of a reaction is controlled by reactor conditions such as milling time, frequency, number of balls, etc., while the number of mechanochemical contacts between particles is controlled by the amounts of reactants, i.e., particle populations. In a reaction involving two or more solids, different reactivities can be obtained at constant reactor conditions by varying the relative amounts of reactants. This can be considered a reactivity phenomena at an intermediate “particle-scale” in between reactor-level “macroscopic” effects and molecular kinetics. This intermediate kinetic scale is unique to the ball-mill reactor and does not exist in homogeneous fluid-phase reactors.

The addition of Fe to PS90 (Figure 5b) and BA to PS50 (Figure 7d) as catalysts achieved an optimal effect at 15 wt % Fe and 5 wt % BA, respectively. In between the optimal composition and the complete absence of additives, this kinetic regime can be understood as analogous to the intrinsic kinetic regime in a traditional heterogeneous packed bed reactor (PBR), where the amount of catalyst present determines the rate of reaction. On the other hand, increasing the amount of additive past the optimal ratio leads to a regime where the catalyst is not being used efficiently, although for reasons different from the transport-limited regime in a PBR. The reduction in monomer yields with increasing catalyst can be explained as a trade-off between catalytic activity and two detrimental effects due to material composition, which is unique to the ball-mill reactor.

First, material response in a portion of reactant powder during each impact depends on the mechanical and thermal properties of that powder,^64^ which are functions of composition—the relative proportions of different material phases present in the reactor. When the relative populations of catalyst and PS particles change, so do these properties. Even in the glassy state, common polymers such as PS tend to possess relatively higher ductility compared to inorganic solids. When a particle of PS is surrounded by more numerous particles of a more brittle and hard material, such as the inorganic catalysts Fe or BA, compression by grinding bodies of this heterogeneous particle ensemble is more likely to lead to plastic deformation of the PS by the harder catalyst particles (which in this context would behave more like rigid bodies relative to the PS), and this outcome—as explained in Section 4.1—is not conducive to monomer production. We may term this a population-induced mechanical integrity effect.

The second effect is that in the ball mill, the probability of physical contact between two types of reactant particles is determined by the composition of the feedstock, more specifically, their absolute population numbers. Denoting catalyst particles as “A”, when the number of A particles increases, the probability of mechanochemical contact of A particles with each other increases while the total number of collisions causing mechanochemical contact stays the same (since milling time and frequency were kept constant). Each grinding surface collision can only act on a small portion of the total particle population, which exacerbates the likelihood of A–A contacts when A particles are in excess of PS. Therefore, even though the desired type of contact for monomer production is between A and PS particles, the greater prevalence of A–A contacts caused by increasing quantities of A in the reactor will at a certain point begin to hinder rather than enhance the reaction rate. Hence, this is termed a particle dilution effect, where the desired type of particle–particle contact that induces depolymerization reactions is “diluted” by undesired particle contact combinations due to relative proportions in the particle population.

Together, the mechanical integrity effect and the dilution effect are kinetic phenomena associated with changes in the composition of the reactant materials that are impacted by grinding surfaces in a single collision. A combination of nonconstructive plastic deformation to the polymer particles by catalyst and statistical dilution when excess catalyst particles are present offers a plausible explanation for the rapid drop in monomer yield with a decreasing proportion of PS past the optimal ratio.

### 4.3 Molecular-Scale Kinetics

Only two prior articles have investigated polyolefin depolymerization in a ball mill with respect to monomer production.^39,40^ Much more literature describes depolymerization and side reactions during thermal pyrolysis,^65^ and we will illustrate that there are intriguing similarities in the mechanisms of polyolefin depolymerization, although these processes occur under different physical conditions.

**Figure 9:**
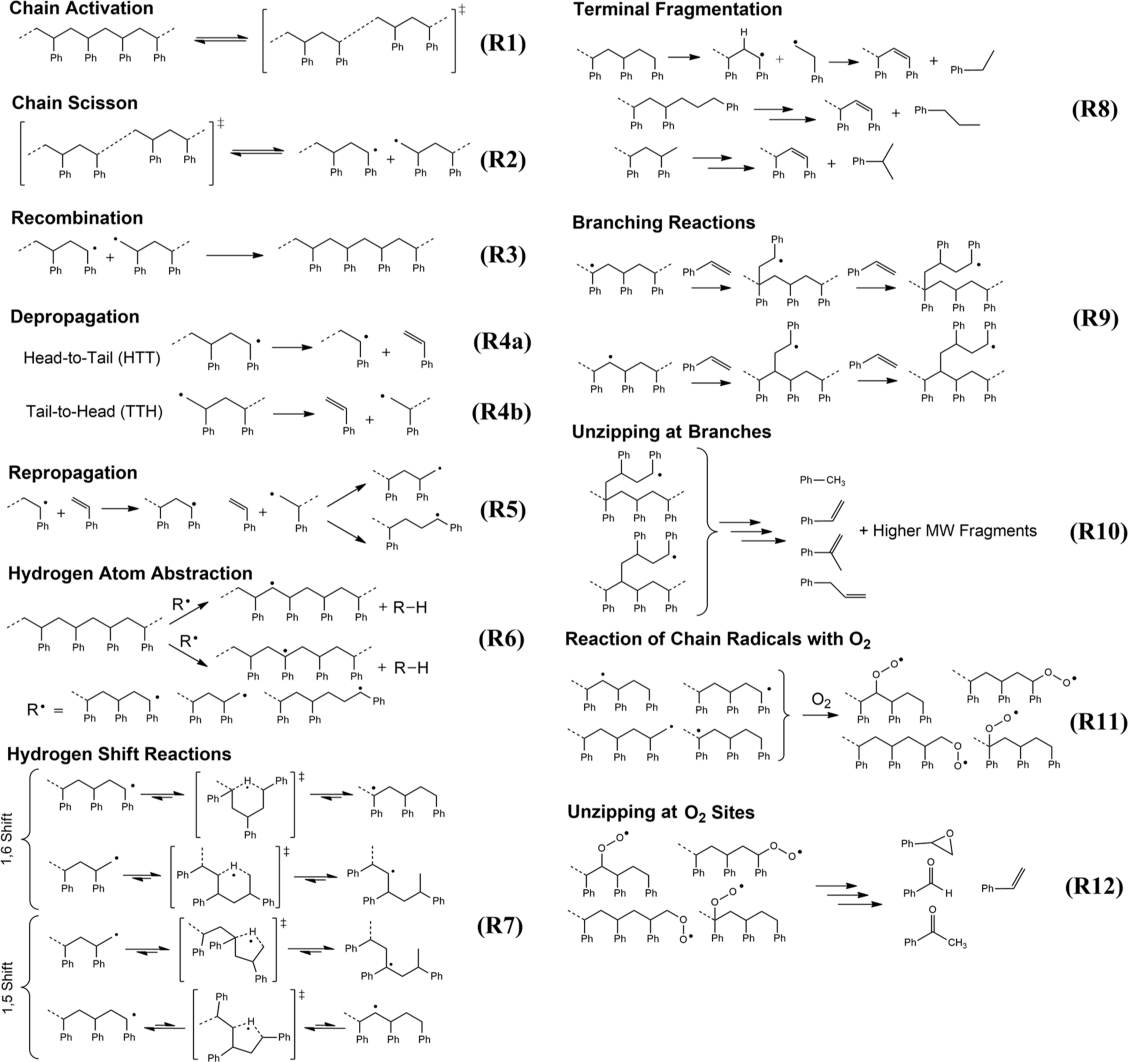
Reaction classes (R1)–(R12) are involved in the depolymerization of PS to monomers. The specific examples illustrated under each heading may be taken as a representative of the broader class of reactions involving structurally related radical species.

Because depolymerization and MW reduction can proceed independently of each other (Section 4.1), it is prudent to first summarize the molecular mechanism of mechanochemical MW reduction in polyolefins, which is chain scission at the backbone C–C bond followed by disproportionation.^66^ As described by Zhurkov et al., application of mechanical force on a polyolefin particle causes bond activation, leading to rupture and the formation of primary scission radicals (Figure 9, R1 and R2).^67^ Subsequently, these radicals can undergo tandem hydrogen abstraction and disproportionation steps with neighboring chains that transfer the radicals away from the locations where they were initially generated. This chain reaction results in microcrack formation within the particle, which culminates in the fracture of the particle into smaller pieces.^68^ When the particles have been fractured to a minimum size achievable by the equipment such that further milling does not lead to further particle breakage, the MW reduction would also level off. Indeed, for PS90, DLS measurements indicated that by 120 min of milling the particle size distribution had stabilized to an average value centered around 10 μm, which coincided with the time at which the MW distribution stabilized to an average around 10,000 g/mol (Figure S6).

The microcrack mechanism explains the formation of radical species but does not include the formation of monomers as observed during milling of PS under N_2_ flow without additional chemical catalysts. However, the formation of styrene from radicalic intermediates is consistent with the reaction network of PS pyrolysis.^69−71^ The key elementary steps in styrene formation are the generation of a pair of chain end radicals by scission, followed by depropagation (the reverse of the propagation step in chain-growth polymerization) (Figure 9, R4a-b).^72^ Depolymerization or “unzipping” is a sequence of consecutive depropagation steps along a chain, proceeding via either the primary or secondary chain end radical. Unzipping a chain can produce any number of styrene molecules up to the degree of polymerization.

Mechanochemical depolymerization of styrene also yielded a number of identified volatile byproducts (Figure 1). In addition, small amounts of high-boiling point products were also detected bt GC–MS, and representative mass spectra are included in Table S3. The appearance of these minor products further illustrates parallels between the underlying mechanisms of mechanochemical depolymerization and pyrolysis. Specific mechanisms for the formation of hydrocarbon products such as toluene, ethylbenzene, allylbenzene, and α-methylstyrene have been comprehensively discussed in the pyrolysis literature and are illustrated in Figure 9.^73−75^ Relevant steps, such as hydrogen atom abstraction, hydrogen shift, and branching by repropagation (Figure 9, R6, R7, and R9, respectively), impart a large number of new moieties onto the polymer—such as quaternary carbons and tertiary carbons not adjacent to any aromatic rings—and thus increase the variety of reactive species in the system. The appearance of the peak at 30 ppm in the ^13^C NMR spectra of milled polymer residues (Figure 4) can be attributed to the new aliphatic carbon environments associated with these new moieties. The location of the peak is consistent with similar carbon environments such as a quaternary β-carbon on neopentylbenzene, a tertiary β-carbon on isobutylbenzene, or a secondary α-carbon on ethylbenzene.^76^

In addition to the hydrocarbon minor products, oxygenates were found among the depolymerization products in all experiments, even when milling was conducted under N_2_ flow after a N_2_ purge (Figure 2b). Since oxygenated monomers were detected even under oxygen-poor conditions, this strongly suggests that the mechanochemical reaction paths in which oxygen species participate to generate monomers are kinetically favored over pathways that involve no oxygen. Increased styrene yield when oxygen content was increased in the gas stream attests to this, although pure O_2_ flow did not improve yield over air flow for PS90, and only marginally for PS50 (Figure 7c). These results indicate that air contains enough oxygen for use in depolymerization and the elementary step in which O_2_ enters the mechanism is not rate-determining.

During pyrolysis reactions, O_2_ can attach to a carbon-centered radical chain end to form a peroxyl radical.^77^ The peroxy radical then abstracts a hydrogen atom, and subsequent elimination of the hydroxy radical leaves a chain-end oxy radical, which is the site of depropagation. Thus, we propose an O_2_-initiated pathway via a peroxy radical intermediate at the metal grinding surfaces as an alternative for depolymerization under mechanochemical conditions. The presence of both carbon- and oxygen-centered radicals in the polymer residue has also been confirmed by the EPR results of Balema et al.^39^ Jung et al.^40^ also detected trace amounts of the oxygenate acetophenone in their products when milling PMS under (closed) air atmosphere, although they dismissed the significance of oxygen toward the depolymerization mechanism by reasoning that the same monomer yield was obtained regardless of whether their reactor was charged with air or an oxygen-free argon atmosphere. We can rationalize this discrepancy on the basis of the low ceiling temperature of PMS, which allows chain activation, scission, and depropagation to all occur spontaneously due to favorable thermodynamics. In the case of comparatively high-ceiling temperature PS, for which spontaneous depolymerization from a carbon-centered radical chain end is not thermodynamically favored at near ambient temperature, depolymerization from a peroxy radical chain end alleviates this deficiency. Since peroxyl radicals are chemically more stable than carbon radicals, O_2_ can be understood to serve the dual role of scission radical stabilizer by converting carbon-centered radicals to peroxyl radicals, thereby retarding recombination reactions in the aftermath of scission. Although oxygen converts reactive scission radicals to chemically less reactive peroxy radicals, the reactivity of the radical site is reoriented toward a more thermodynamically favored mechanochemical mechanism.

In the mechanism proposed by Balema et al.,^39^ a peroxy chain end radical complexes with an unspecified metal species to activate the chain for depolymerization. In this study, that metal species was found to be Fe and—to a lesser extent—Cr, since styrene, the yield was enhanced by those two metals, while Ni appeared not to facilitate depolymerization via this oxygen-assisted mechanism, as reflected in a reduced styrene yield (Figure 5a). In this study, it was also observed that Cr loses its ability to enhance depolymerization during milling in air, which can be attributed to the tendency of Cr to rapidly form a passivating oxide layer on its surface,^78^ which is inactive. Fe, on the other hand, does not form such a layer as readily^79^ and preserves some catalytic activity when the finite oxygen supply in a closed reactor is replaced by a gas phase of constant oxygen concentration in the open reactor configuration. We would also expect metal oxide powders to be poorer catalyst materials for depolymerization than their metals. For instance, iron oxide (Fe_2_O_3_) is the fully oxidized form of iron; hence, the observed suppression in monomer yield in its presence is logical.

The collapse of the styrene concentration after milling beyond 160 min in a closed vessel can be attributed to repropagation and branching reactions (Figure 9, R5 and R9), which consume styrene monomers. The NMR peak at attributed to branching points at 30 ppm indeed increased between 160 and 240 min (Figure 4), coinciding with the collapse in styrene concentration in the closed reactor during that time frame (Figure 2a). However, when that collapse was avoided using air flow, the magnitude of the peak after 360 min was only comparable to the same after 160 min in the closed vessel.

The initial monomer production rate for PS50 was 15 times lower compared to PS90 (Figure 2a vs Figure 7a) under closed conditions. Under open conditions, PS50 also saw a small increase in yield when oxygen concentration in the flow gas was increased from none to air to pure O_2_ (Figure 7c), which suggests that unlike PS90, for depolymerization to proceed in this material, the abundance of O_2_ is more important, likely due to a scarcity of scission radicals that a given O_2_ molecule in a gas phase of air can encounter on a per unit time basis. When the entire gas phase is pure O_2_, this scarcity is alleviated so there is a greater likelihood that the few radicals that do form are captured efficiently.

The most significant difference in reactivity between PS90 and PS50 was the apparent lack of a chemical effect of BA on the former. The oligomeric fraction of PS50 did not undergo MW reduction under the mechanochemical conditions of this study (see Figure S1b,d), but the combination of BA with this oligomeric fraction in the closed reactor made the higher MW PS present in the material more reactive while not reducing the MW of the oligomers themselves. Even so, the styrene yield from PS50 with BA was still 3 times lower than that from PS90. Also interesting is the finding that BA loses much of its effectiveness at enhancing monomer production when paired with any kind of gas flow (Figure 7c). These two results imply a unique interaction of BA with both oligomeric PS and accumulated monomers inside the sealed reactor environment that increases monomer production, with one possibility being that BA uses some of the monomers to activate the oligomeric PS as chain transfer agents, which increases radical concentrations. The detriment of combining BA with air or O_2_ flow for PS50 may also be rationalized as the degradation of BA by O_2_ (i.e., calcination^80^). The closed reactor with a finite and decreasing O_2_ content was insufficient to interfere with BA’s unique activity with PS50, but in the open reactor with a constant O_2_ atmosphere any beneficial effect of BA was likely overshadowed.

Evidently, BA served a more complex role in the depolymerization mechanisms by simultaneously preserving the constant rate of styrene production and tilting selectivity gradually in favor of minor products (Figure 7b). An implication of this is that BA may in part be a “reagent” that is consumed slowly, as indicated by this shift in selectivity.

## 5 Conclusions

In this article, a variety of kinetic phenomena associated with mechanochemical PS depolymerization were elucidated. These phenomena can be associated with three characteristic length scales of different magnitudes in the system.^81^

The first length scale is the molecular or mechanistic scale associated with free radical chemical events occurring at specific moieties on the PS chains. These phenomena occur at scales on the order of Ångstroms to nanometers on or near the surfaces of the reactant particles. Unzipping of chains—driven by transient conditions in the ball mill that render depolymerization favorable thermodynamically—suffices to explain styrene formation directly, and the appearance of small quantities of other compounds—hydrocarbons and oxygenates—can be construed as products of unzipping at structural moieties introduced onto the PS chains by the same mechanisms present at thermal pyrolysis conditions. Depolymerization was enhanced by molecular oxygen via a mechanism similar to that involved in oxidative pyrolysis. The presence of repolymerization justifies implementing a flowing gas stream to continuously remove volatile monomers, which serves the dual purpose of replenishing the oxygen supply in the reactor and maintaining a low average reactor concentration of styrene, thereby suppressing repolymerization.

Above the molecular scale is the particle or material scale, which is on the order of micrometers. This scale encompasses kinetic effects arising from the composition-dependent material response of the reactant particles to mechanical force and the statistics of heterogeneous particle–particle interactions. At this scale, it was demonstrated using Fe and BA powders that an optimal ratio of polymer to catalyst could be established in the trade-off between enhanced monomer production due to catalytic activity and reactant composition-dependent mechanical and mixing effects detrimental to depolymerization.

Finally, kinetics at the reactor scale make up the third group of phenomena. Manifested at this scale are kinetic characteristics of depolymerization that depend directly upon reactor-averaged parameters such as mechanical energy input (tuned through parameters such as mill frequency, ball mass, etc.), concentrations of intermediate species and products, reactor geometry, and macroscopic temperature. At this scale, it was shown that mechanical energy supply is simultaneously responsible for chain scission and depolymerization/monomer production phenomena, with the two in proportion to each other when normalized on a per-polymer-chain basis during the early stages of milling. Increasing the mechanical energy input increases the depolymerization when competing temperature-induced mechanical effects are absent. Conditions that create increased collision energy dissipation lead to a greater temperature rise. When considered alongside the known temperature-dependent mechanical properties of solid PS, especially in the vicinity of its glass transition temperature range, accumulated heat can lead to the softening of polymer particles and other changes in their mechanical properties that are detrimental to depolymerization.

Undoubtedly, solid-state depolymerization of PS in the vibratory ball-mill reactor reveals a rich tapestry of unique kinetic phenomena and suggests promising applications for the chemical recycling of polyolefins by using mechanochemistry.

## Supporting Information Available

Numerical values of monomer yield for time-series experiments in tabulated form, molecular weight distributions, total ion chromatogram of representative experiment, individual mass spectra of monomeric products, NMR spectra of select samples, ATR-FTIR spectrum of PS powder, and particle size distribution for time-series experiments (PDF)

## Abbreviations

BA: boric acid
DLS: dynamic light scattering
GC: gas chromatography
GPC: gel permeation chromatography
MS: mass spectrometry
MW: molecular weight
NMR: nuclear magnetic resonance
PMS: poly(α-methylstyrene)
PS: poly(styrene)
