# Supplementary material for: Kinetic Phenomena in Mechanochemical Depolymerization of Poly(styrene)
Source: ACS Sustain Chem Eng. 2023 Dec 16;12(1):178–91. doi: 10.1021/acssuschemeng.3c05296 (PMC10777454; doi:10.1021/acssuschemeng.3c05296)
Supplement: Supplementary file 1 — sc3c05296_si_001.pdf [file sc3c05296_si_001.pdf]

Supplementary Information

Kinetic Phenomena in Mechanochemical Depolymerization of Poly(Styrene)

Yuchen Chang[1], Sylvie J. Blanton[1], Ralph Andraos[1], Van Son Nguyen[1,2],
Charles L. Liotta[3], F. Joseph Schork[1] and Carsten Sievers[1]*
[1]School of Chemical & Biomolecular Engineering, Georgia Institute of Technology, Atlanta, Georgia, 30332 (U.S.A.)
[2]Department of Chemistry, Technical University of Munich, Lichtenbergstrasse 4, 85748 (Germany)
[3]School of Chemistry & Biochemistry, Georgia Institute of Technology, Atlanta, Georgia, 30332 (U.S.A.)
*carsten.sievers@chbe.gatech.edu

Number of pages: 21
Number of figures: 20
Number of tables: 3

S.A. Monomer Yields

Table S1: Tabulated yields versus milling time for monomers shown in Figure 1, with starting materials PS50 and PS90 milled under prescribed conditions (cl. = closed reactor with air atmosphere, op.n = open reactor with N_2 flow, op.a = open reactor with air flow). For open reactor yields, E = effluent portion and R = reactor portion. Monomer designations are M1 = styrene, M2 = toluene, M4 = ethylbenzene, M5 = allylbenzene, M6 = α-methylstyrene, M9 = styrene oxide, M10 = benzaldehyde, M11 = acetophenone. M3 (= 1-propenylbenze), M7 (= n-propylbenzene) and M8 (= cumene) are only detected in trace quantities and across all experimental conditions and therefore are omitted in the table. BA = boric acid.

		Monomer yields (mg/g PS)							
Milling time (min.)		20	40	60	80	120	160	200	240
M1	PS90 cl.	3.13 ±0.01	5.38 ±0.51	11.23 ±0.66	15.79 ±3.03	28.26 ±5.48	34.80 ±2.42	7.17 ±2.08	5.78 ±0.75
	PS90+M1 cl.	13.07 ±0.14	15.91 ±0.48	- -	3.18 ±0.17	- -	- -	- -	- -
	PS50 cl.	0.31 ±0.10	0.78 ±0.14	- -	1.03 ±0.20	1.31 ±0.20	1.66 ±0.16	2.86 ±0.27	3.80 ±0.84
	PS50+BA cl.	1.32 ±0.16	2.65 ±1.04	- -	5.61 ±0.51	7.89 ±0.40	10.23 ±1.90	13.98 ±2.79	17.44 ±0.65
	PS90 op.n (R)	0.55 ±0.03	1.04 ±0.02	1.55 ±0.07	2.12 ±0.23	2.11 ±0.10	4.30 ±0.35	4.67 ±1.63	6.46 ±0.17
	PS90 op.n (E)	0.92 ±0.02	1.15 ±0.08	1.89 ±0.14	2.51 ±0.08	4.83 ±0.02	4.64 ±0.29	8.31 ±1.29	12.15 ±0.01
	PS90 op.a (R)	1.31 ±0.20	4.84 ±0.09	9.00 ±0.45	10.05 ±1.74	14.46 ±0.03	10.91 ±0.17	12.41 ±0.57	18.52 ±1.84
	PS90 op.a (E)	1.66 ±0.16	1.28 ±0.47	4.01 ±0.10	7.83 ±0.57	15.20 ±0.08	25.39 ±1.55	31.81 ±6.37	34.35 ±2.87
M2	PS90 cl.	0.03 ±0.01	0.01 ±0.03	0.13 ±0.03	0.20 ±0.06	0.44 ±0.07	0.55 ±0.02	0.49 ±0.22	0.46 ±0.21
	PS90+M1 cl.	0.00 ±0.00	0.02 ±0.03	- -	0.00 ±0.00	- -	- -	- -	- -
	PS50 cl.	0.00 ±0.00	0.00 ±0.00	- -	0.00 ±0.00	0.03 ±0.03	0.00 ±0.00	0.01 ±0.02	0.03 ±0.03
	PS50+BA cl.	0.00 ±0.00	0.00 ±0.00	- -	0.01 ±0.03	0.11 ±0.09	0.13 ±0.12	0.23 ±0.05	0.39 ±0.02
	PS90 op.n (R)	0.00 ±0.00	0.00 ±0.00	0.06 ±0.04	0.11 ±0.03	0.05 ±0.01	0.08 ±0.00	0.13 ±0.11	0.10 ±0.01
	PS90 op.n (E)	0.06 ±0.05	0.03 ±0.01	0.24 ±0.03	0.22 ±0.01	0.52 ±0.01	0.54 ±0.02	0.92 ±0.15	1.23 ±0.01
	PS90 op.a (R)	0.03 ±0.03	0.04 ±0.01	0.03 ±0.01	0.08 ±0.01	0.08 ±0.00	0.00 ±0.00	0.00 ±0.00	0.06 ±0.02
	PS90 op.a (E)	0.00 ±0.00	0.07 ±0.09	0.15 ±0.02	0.29 ±0.03	0.49 ±0.00	0.52 ±0.14	0.63 ±0.21	0.69 ±0.22

M4	PS90 cl.	0.02 ±0.00	0.01 ±0.02	0.05 ±0.04	0.20 ±0.10	0.43 ±0.07	0.48 ±0.07	0.54 ±0.17	0.55 ±0.16
	PS90+M1 cl.	0.00 ±0.00	0.02 ±0.03	- -	0.00 ±0.00	- -	- -	- -	- -
	PS50 cl.	0.00 ±0.00	0.00 ±0.00	- -	0.00 ±0.00	0.00 ±0.00	0.00 ±0.00	0.03 ±0.03	0.08 ±0.00
	PS50+BA cl.	0.00 ±0.00	0.05 ±0.09	- -	0.11 ±0.10	0.31 ±0.01	0.23 ±0.20	0.52 ±0.07	0.65 ±0.04
	PS90 op.n (R)	0.00 ±0.00	0.00 ±0.00	0.07 ±0.03	0.05 ±0.03	0.05 ±0.02	0.16 ±0.03	0.18 ±0.16	0.17 ±0.03
	PS90 op.n (E)	0.02 ±0.02	0.04 ±0.01	0.12 ±0.05	0.10 ±0.02	0.31 ±0.03	0.41 ±0.01	0.70 ±0.02	0.92 ±0.01
	PS90 op.a (R)	0.00 ±0.00	0.02 ±0.03	0.06 ±0.01	0.09 ±0.02	0.14 ±0.00	0.04 ±0.03	0.00 ±0.00	0.12 ±0.07
	PS90 op.a (E)	0.00 ±0.00	0.02 ±0.03	0.08 ±0.02	0.14 ±0.01	0.34 ±0.02	0.46 ±0.08	0.55 ±0.13	0.61 ±0.15
M5	PS90 cl.	0.03 ±0.02	0.00 ±0.00	0.33 ±0.08	0.41 ±0.39	0.68 ±0.59	0.00 ±0.00	0.24 ±0.21	0.47 ±0.56
	PS90+M1 cl.	0.00 ±0.00	0.00 ±0.00	- -	0.21 ±0.18	- -	- -	- -	- -
	PS50 cl.	0.00 ±0.00	0.03 ±0.03	- -	0.03 ±0.03	0.05 ±0.04	0.08 ±0.01	0.13 ±0.02	0.19 ±0.02
	PS50+BA cl.	0.00 ±0.00	0.00 ±0.00	- -	0.00 ±0.00	0.00 ±0.00	0.00 ±0.00	0.00 ±0.00	0.94 ±0.00
	PS90 op.n (R)	0.02 ±0.02	0.17 ±0.09	0.02 ±0.03	0.16 ±0.14	0.07 ±0.01	0.40 ±0.03	0.21 ±0.18	0.52 ±0.02
	PS90 op.n (E)	0.03 ±0.01	0.05 ±0.01	0.00 ±0.00	0.13 ±0.01	0.01 ±0.01	0.35 ±0.07	0.39 ±0.35	0.68 ±0.01
	PS90 op.a (R)	0.05 ±0.04	0.11 ±0.03	0.23 ±0.03	0.00 ±0.00	0.00 ±0.00	0.00 ±0.00	0.00 ±0.00	2.39 ±1.95
	PS90 op.a (E)	0.08 ±0.01	0.00 ±0.00	0.06 ±0.02	0.11 ±0.02	0.11 ±0.19	0.48 ±0.50	0.35 ±0.61	0.69 ±0.34
M6	PS90 cl.	0.00 ±0.01	0.02 ±0.00	0.02 ±0.03	0.10 ±0.04	0.23 ±0.06	0.25 ±0.04	0.11 ±0.06	0.07 ±0.06
	PS90+M1 cl.	0.00 ±0.00	0.01 ±0.01	- -	0.00 ±0.00	- -	- -	- -	- -
	PS50 cl.	0.05 ±0.08	0.03 ±0.03	- -	0.06 ±0.01	0.05 ±0.05	0.09 ±0.02	0.13 ±0.03	0.20 ±0.04
	PS50+BA cl.	0.12 ±0.10	0.10 ±0.08	- -	0.37 ±0.04	0.41 ±0.02	0.59 ±0.10	0.80 ±0.16	0.92 ±0.10
	PS90 op.n (R)	0.00 ±0.00	0.00 ±0.00	0.01 ±0.01	0.01 ±0.02	0.01 ±0.02	0.08 ±0.01	0.08 ±0.05	0.12 ±0.00
	PS90 op.n (E)	0.00 ±0.00	0.00 ±0.00	0.01 ±0.01	0.00 ±0.00	0.03 ±0.00	0.04 ±0.00	0.03 ±0.05	0.11 ±0.00

	PS90<br>op.a (R)	0.05<br>±0.05	0.01<br>±0.01	0.05<br>±0.01	0.07<br>±0.03	0.13<br>±0.00	0.07<br>±0.06	0.12<br>±0.05	0.21<br>±0.03
	PS90<br>op.a (E)	0.09<br>±0.02	0.00<br>±0.00	0.00<br>±0.00	0.02<br>±0.02	0.02<br>±0.04	0.13<br>±0.04	0.19<br>±0.03	0.06<br>±0.10
M9	PS90<br>cl.	0.08<br>±0.01	0.29<br>±0.11	0.63<br>±0.23	1.61<br>±0.60	1.70<br>±0.63	2.04<br>±0.69	1.65<br>±1.70	1.08<br>±1.00
	PS90+M1<br>cl.	0.53<br>±0.12	1.51<br>±0.29	-<br>-	3.18<br>±1.27	-<br>-	-<br>-	-<br>-	-<br>-
	PS50<br>cl.	0.00<br>±0.00	0.08<br>±0.03	-<br>-	0.10<br>±0.01	0.13<br>±0.09	0.12<br>±0.00	0.25<br>±0.06	0.34<br>±0.13
	PS50+BA<br>cl.	0.01<br>±0.02	0.01<br>±	-<br>-	0.14<br>±0.13	0.14<br>±0.12	0.21<br>±0.19	0.38<br>±0.10	1.22<br>±0.21
	PS90<br>op.n (R)	0.01<br>±0.01	0.00<br>±0.00	0.01<br>±0.02	0.11<br>±0.13	0.03<br>±0.04	0.28<br>±0.08	0.14<br>±0.06	0.31<br>±0.01
	PS90<br>op.n (E)	0.00<br>±0.00	0.00<br>±0.00	0.00<br>±0.00	0.00<br>±0.00	0.00<br>±0.00	0.00<br>±0.00	0.01<br>±0.01	0.00<br>±0.00
	PS90<br>op.a (R)	0.13<br>±0.09	0.20<br>±0.01	0.33<br>±0.03	0.75<br>±0.12	1.26<br>±0.05	0.95<br>±0.12	1.22<br>±0.26	1.99<br>±0.54
	PS90<br>op.a (E)	0.12<br>±0.00	0.00<br>±0.00	0.00<br>±0.00	0.02<br>±0.02	0.02<br>±0.03	0.82<br>±0.89	0.73<br>±1.02	0.06<br>±0.11
M10	PS90<br>cl.	0.27<br>±0.05	0.69<br>±0.17	1.21<br>±0.22	2.50<br>±0.83	3.85<br>±0.36	5.02<br>±0.96	3.38<br>±3.13	2.70<br>±1.76
	PS90+M1<br>cl.	0.97<br>±0.16	2.14<br>±0.44	-<br>-	4.80<br>±2.74	-<br>-	-<br>-	-<br>-	-<br>-
	PS50<br>cl.	0.00<br>±0.00	0.18<br>±0.02	-<br>-	0.20<br>±0.03	0.21<br>±0.07	0.29<br>±0.01	0.38<br>±0.08	0.49<br>±0.16
	PS50+BA<br>cl.	0.07<br>±0.06	0.25<br>±0.22	-<br>-	0.48<br>±0.45	1.16<br>±0.13	0.94<br>±0.83	2.22<br>±0.47	3.69<br>±0.66
	PS90<br>op.n (R)	0.03<br>±0.04	0.07<br>±0.02	0.12<br>±0.06	0.07<br>±0.12	0.22<br>±0.03	0.23<br>±0.03	0.36<br>±0.31	0.26<br>±0.01
	PS90<br>op.n (E)	0.00<br>±0.00	0.04<br>±0.02	0.12<br>±0.02	0.00<br>±0.00	0.12<br>±0.17	0.02<br>±0.00	0.29<br>±0.24	0.14<br>±0.00
	PS90<br>op.a (R)	0.21<br>±0.07	0.60<br>±0.07	0.95<br>±0.05	2.02<br>±0.22	3.21<br>±0.04	2.61<br>±0.20	3.67<br>±0.11	2.57<br>±2.58
	PS90<br>op.a (E)	0.29<br>±0.01	0.01<br>±0.01	0.05<br>±0.02	0.19<br>±0.06	0.59<br>±0.18	2.31<br>±2.15	2.91<br>±1.86	0.91<br>±0.27
M11	PS90<br>cl.	0.00<br>±0.00	0.00<br>±0.00	0.04<br>±0.06	0.15<br>±0.16	0.22<br>±0.21	0.00<br>±0.00	0.29<br>±0.21	0.34<br>±0.17
	PS90+M1<br>cl.	0.00<br>±0.00	0.00<br>±0.00	-<br>-	0.09<br>±0.08	-<br>-	-<br>-	-<br>-	-<br>-
	PS50<br>cl.	0.00<br>±0.00	0.03<br>±0.03	-<br>-	0.03<br>±0.03	0.05<br>±0.04	0.08<br>±0.01	0.09<br>±0.04	0.13<br>±0.05
	PS50+BA<br>cl.	0.00<br>±0.00	0.00<br>±0.00	-<br>-	0.00<br>±0.00	0.00<br>±0.00	0.00<br>±0.00	0.00<br>±0.00	0.00<br>±0.00

	PS90 op.n (R)	0.00 ±0.00	0.00 ±0.00	0.00 ±0.00	0.01 ±0.02	0.00 ±0.00	0.07 ±0.02	0.03 ±0.03	0.08 ±0.01
	PS90 op.n (E)	0.00 ±0.00	0.00 ±0.00	0.00 ±0.00	0.00 ±0.00	0.00 ±0.00	0.00 ±0.00	0.00 ±0.00	0.00 ±0.00
	PS90 op.a (R)	0.05 ±0.04	0.00 ±0.00	0.03 ±0.01	0.09 ±0.08	0.24 ±0.01	0.19 ±0.01	0.33 ±0.02	0.52 ±0.15
	PS90 op.a (E)	0.08 ±0.01	0.00 ±0.00	0.00 ±0.00	0.00 ±0.00	0.00 ±0.00	0.15 ±0.26	0.16 ±0.28	0.01 ±0.01

S.B. Gel Permeation Chromatography (GPC)

Experiments to determine molecular weights (MW) of solid residues were performed on a Tosoh EcoSEC HLC-8320GPC equipped with TSKgel SuperMultipore HZ-M column, internal refractive index detector (RID) and Wyatt Technology DAWN8+ dynamic light scattering detector (DLS), operating at 40°C. The eluent was chloroform containing 0.3% triethylamine at a flow rate of 0.45 mL/min.

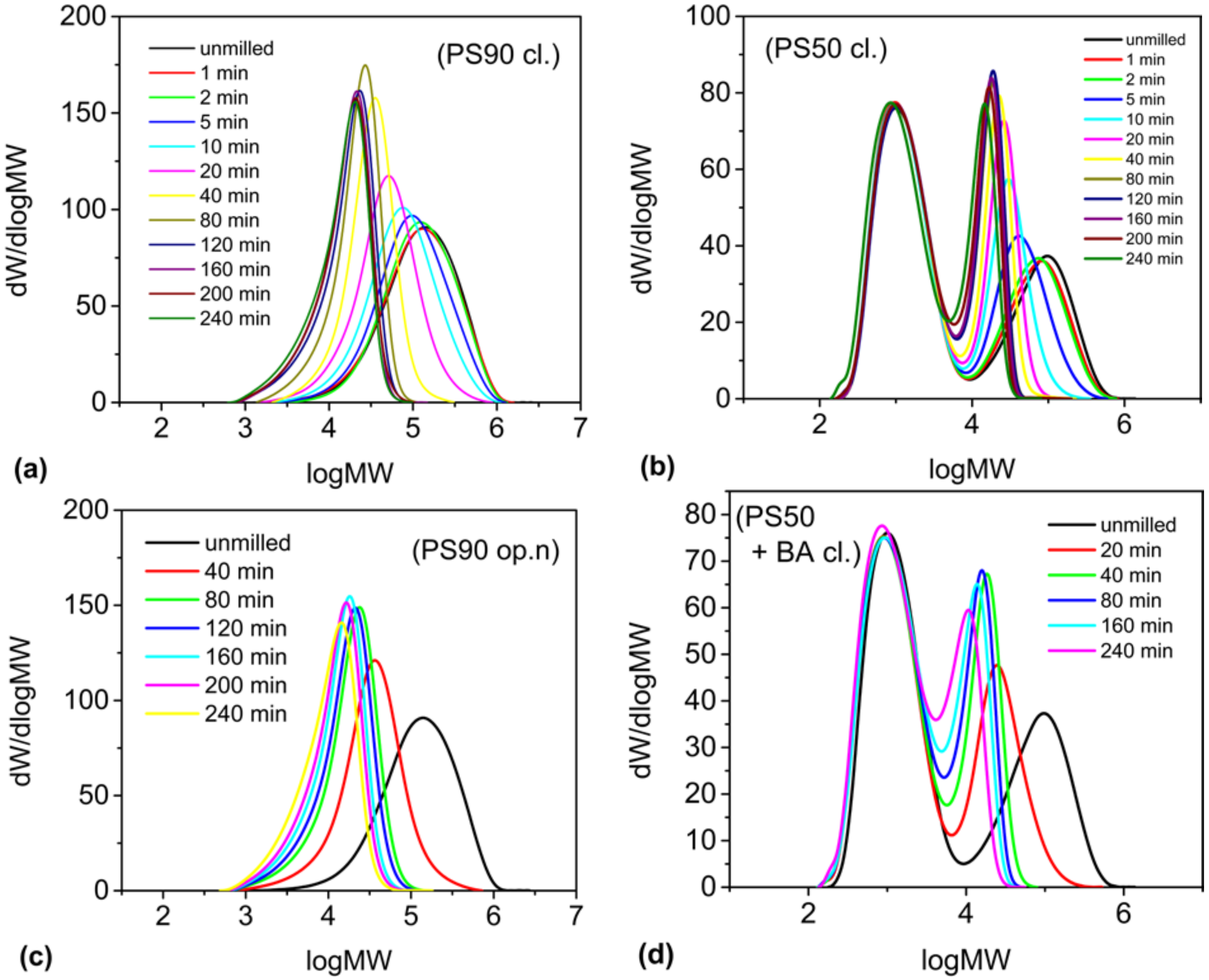

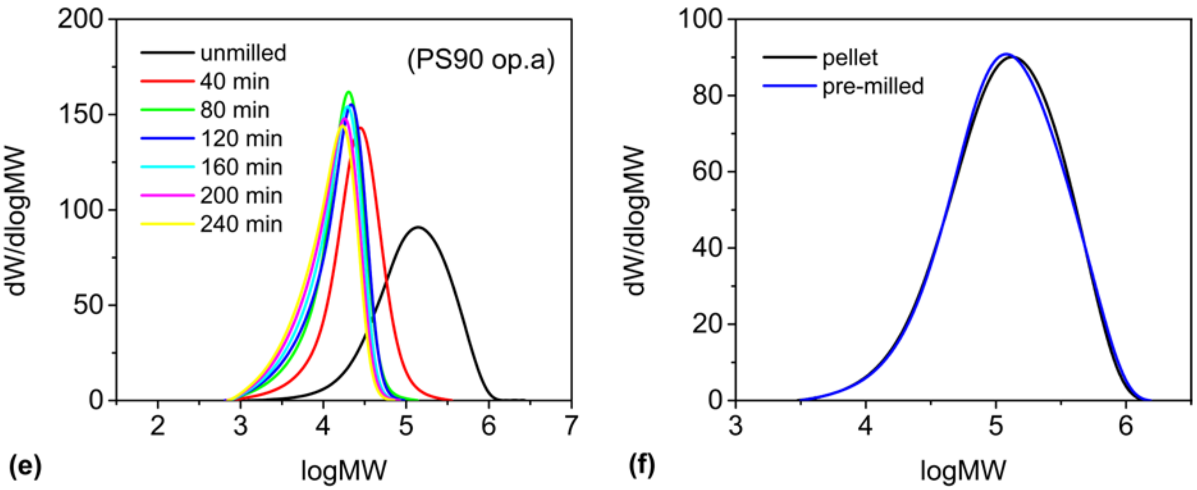

Figure S1: (a)–(e) Molecular weight distributions of residues for different milling times and reactor conditions, with “unmilled” denoting the pre-milled PS90 starting material, (f) molecular weight distributions of uncrushed PS90 pellet and pre-milled starting material. dW = differential weight fraction; MW = molecular weight.

S.C. Gas Chromatography-Mass Spectrometry (GC-MS)

Experiments were performed on an Agilent 8890 GC equipped with HP-5MS UI capillary column coupled to a 5977B GC/MSD operating on electron impact mode. The carrier gas was helium at 1 mL/min. Liquid fraction samples were analyzed to obtain mass spectra of each compound present in the mixture for structure verification.

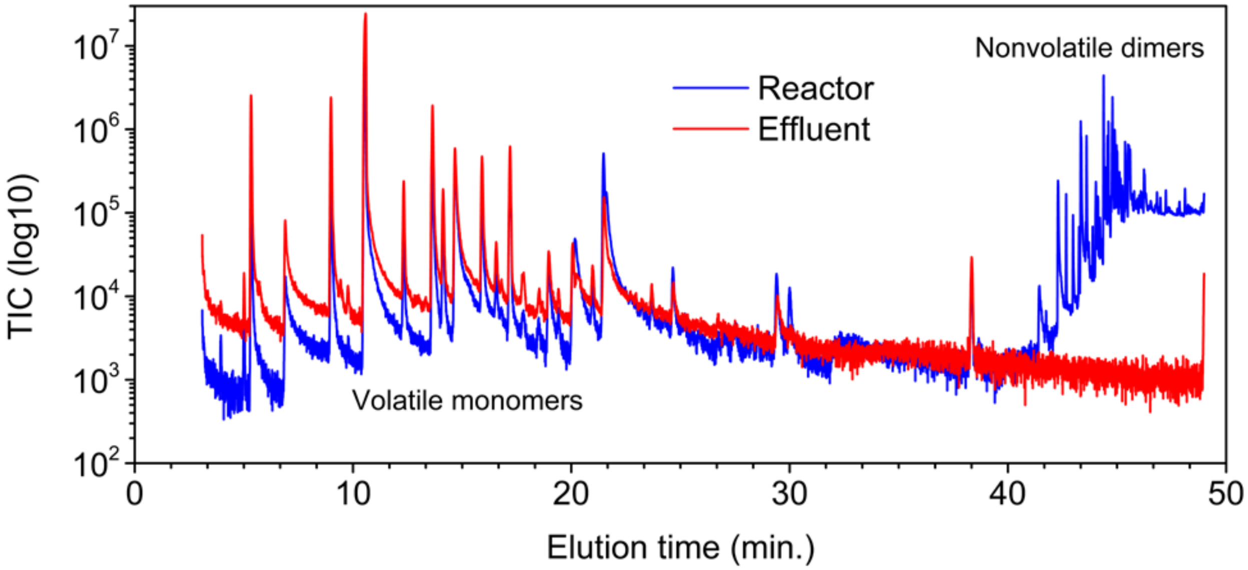

Figure S2: Total ion chromatogram of representative reactor and effluent wash solutions.

Table S2: Mass Spectra (red) of individual peaks from ion chromatogram exhibited with corresponding compound library match (blue) obtained using NIST MS Search version 2.3.

Toluene	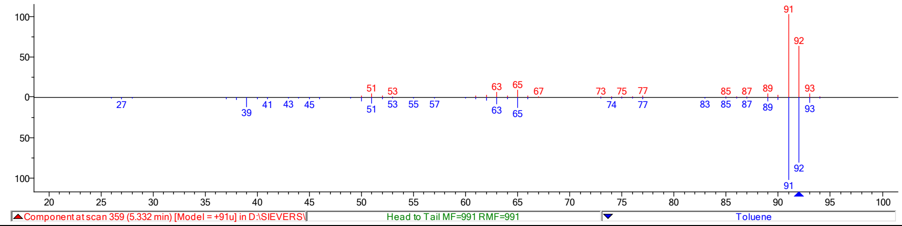	M2
Ethylbenzene	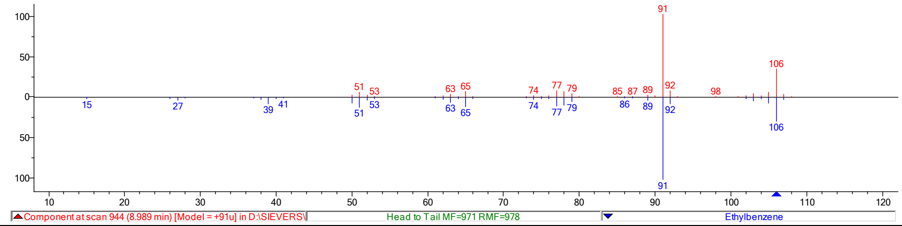	M4
Styrene	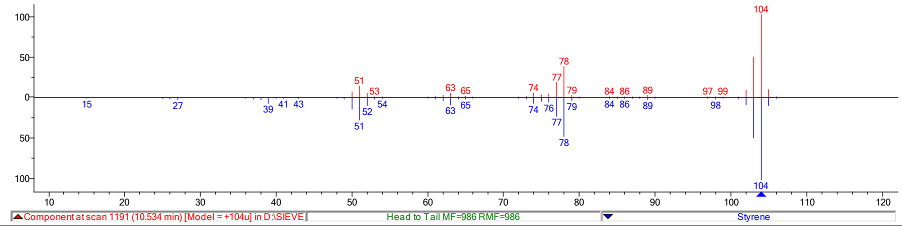	M1
Cumene [(1-methylethyl)-benzene]	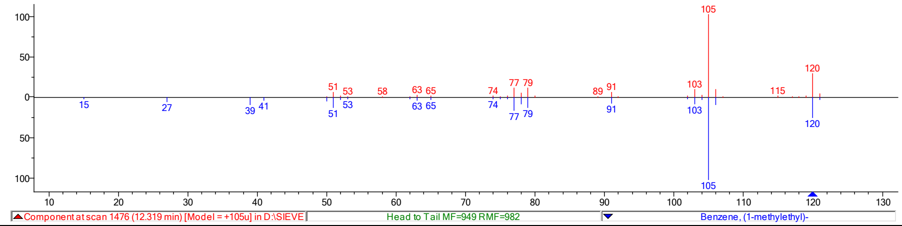	M8
Allylbenzene [2-propenyl-benzene]	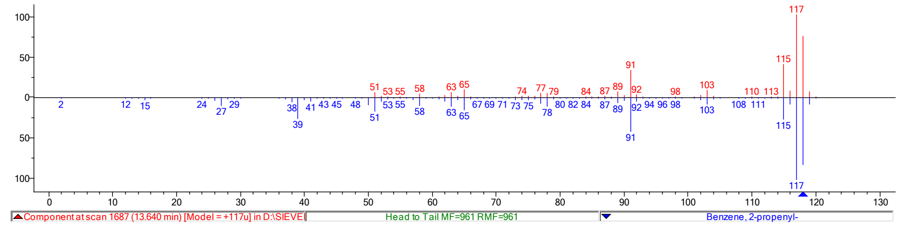	M5
n-Propyl benzene	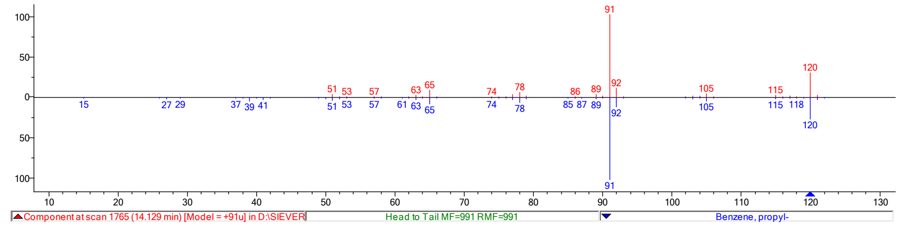	M7
Benzaldehyde	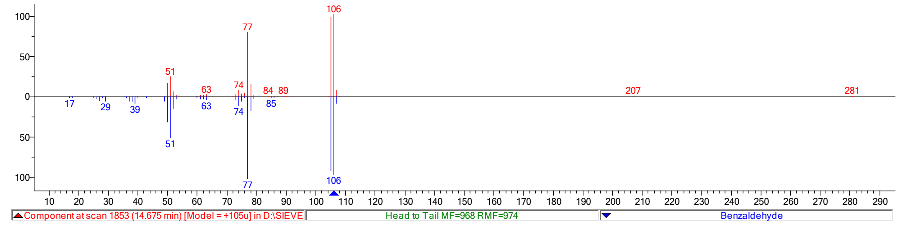	M10

α-Methyl styrene	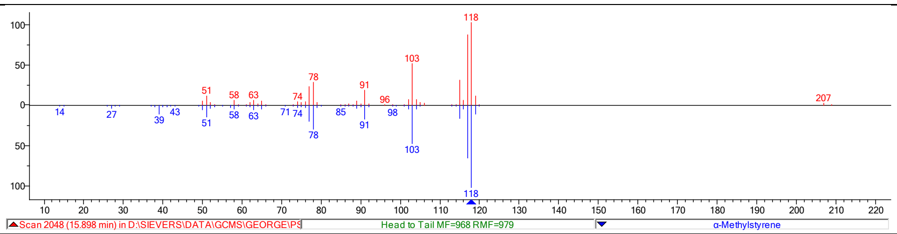	M6
1-propenyl benzene	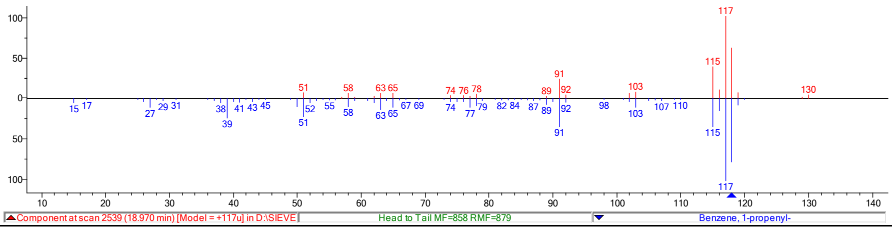	M3
Styrene oxide [phenyl-oxirane]	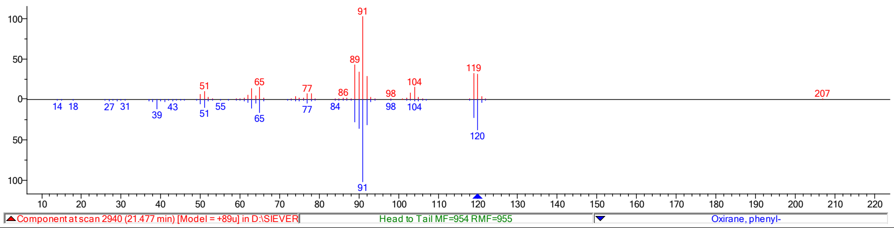	M9
Acetophenone	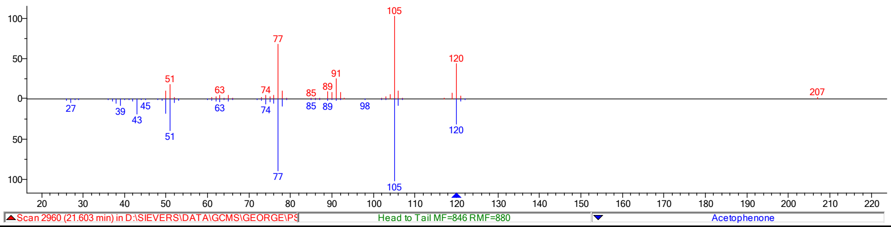	M11

Table S3: Examples of methanol-soluble high-boiling point products detected in GC-MS and suggested molecular structure.

<table>
<tr><th>Mass spectrum</th><th>Suggested structure</th></tr>
<tr><td>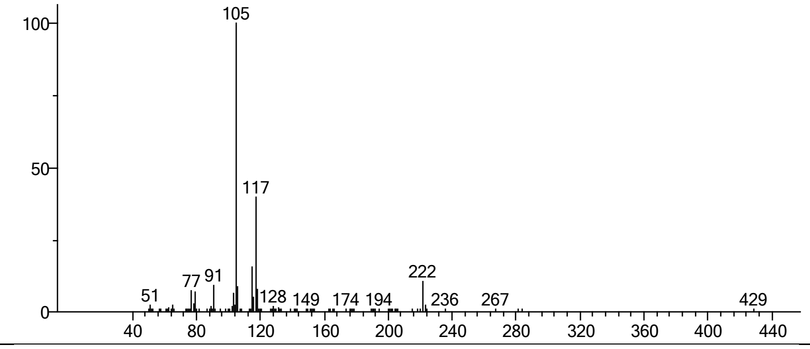
</td><td>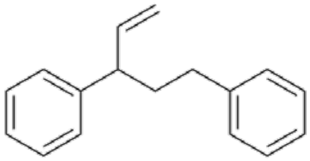
MW = 222 Da</td></tr>
<tr><td>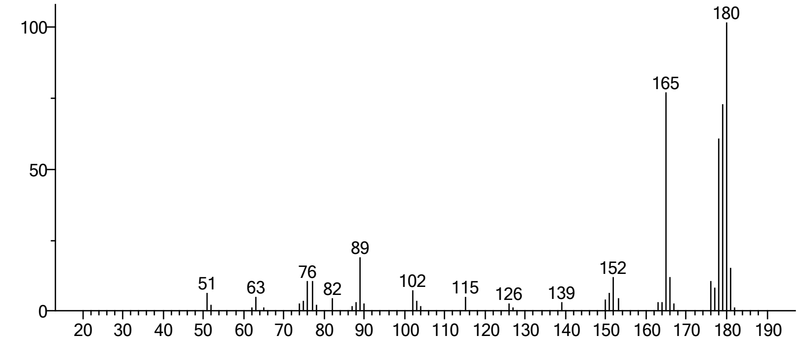
</td><td>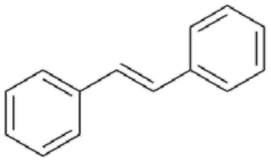
MW = 180 Da</td></tr>
<tr><td>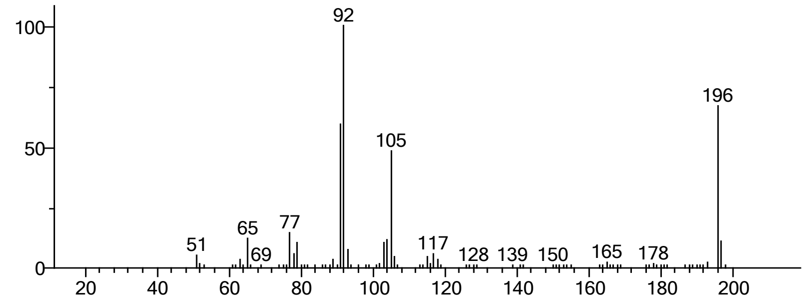
</td><td>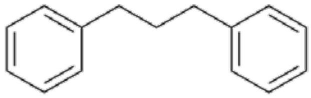
MW = 196 Da</td></tr>
<tr><td>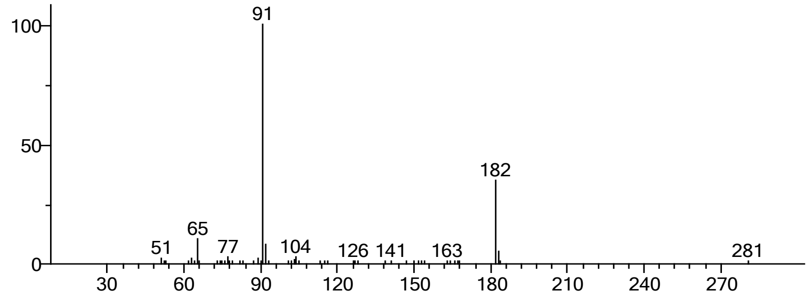
</td><td>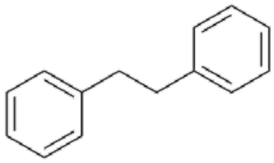
MW = 182 Da</td></tr>
</table>

S.D. Nuclear Magnetic Resonance (NMR) Spectroscopy

^{1}H and ^{13}C NMR spectroscopy experiments were performed on a Bruker AV3 400 MHz NMR spectrometer. Experiments were conducted on both liquid and solid fraction samples prepared in chloroform-d ($CDCl_3$) containing 0.03% (v/v) tetramethylsilane (TMS). For liquid samples, 1 mL of liquid from effluent gas trap was collected directly. For solid samples, 100 mg of sample was dissolved in 1.2 mL of $CDCl_3$ and passed through a 0.2 µm PTFE syringe filter. ^{1}H spectra were collected in the range -3.8 ppm < δ_H < 16.2 ppm with 16 scans per spectrum and calibrated to internal TMS at δ_H = 0.0 ppm. ^{13}C spectra were collected in the range -20 ppm < δ_C < 220 ppm with 1024 scans per spectra and calibrated to the $CDCl_3$ triplet centered at δ_C = 77.23 ppm.

Figure titles prefaced by an asterisk (*) indicate that the corresponding spectra also appear in Figure 4 of the main text in truncated form.

S.D1. ^{13}C NMR

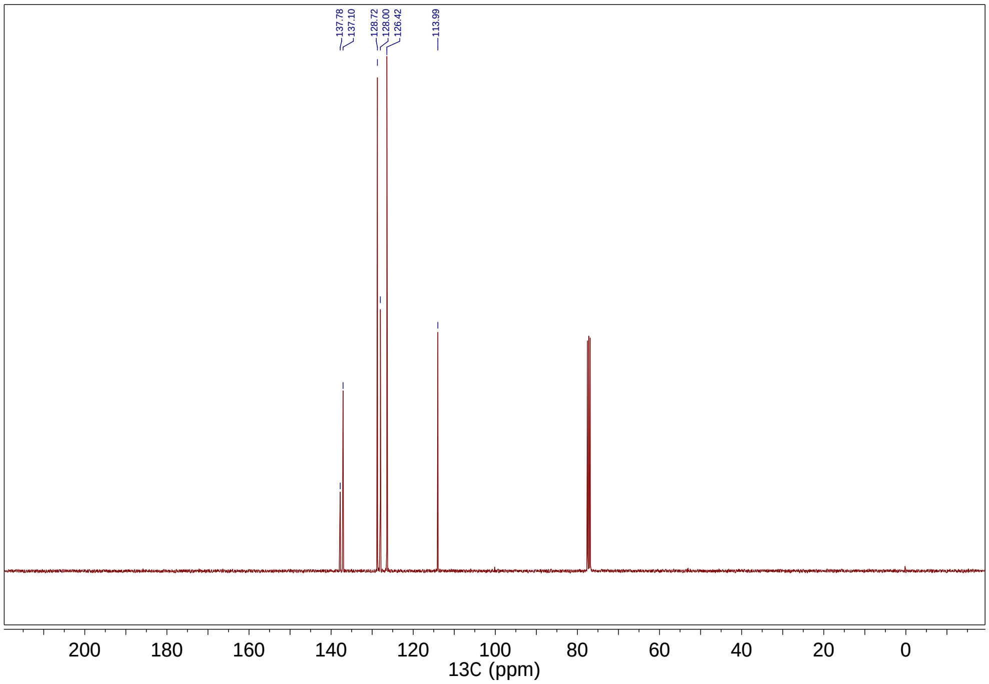

*Figure S3A: Styrene standard

^{13}C NMR (101 MHz, $CDCl_3$) δ 137.78, 137.09, 128.71, 127.99, 126.41, 113.99.

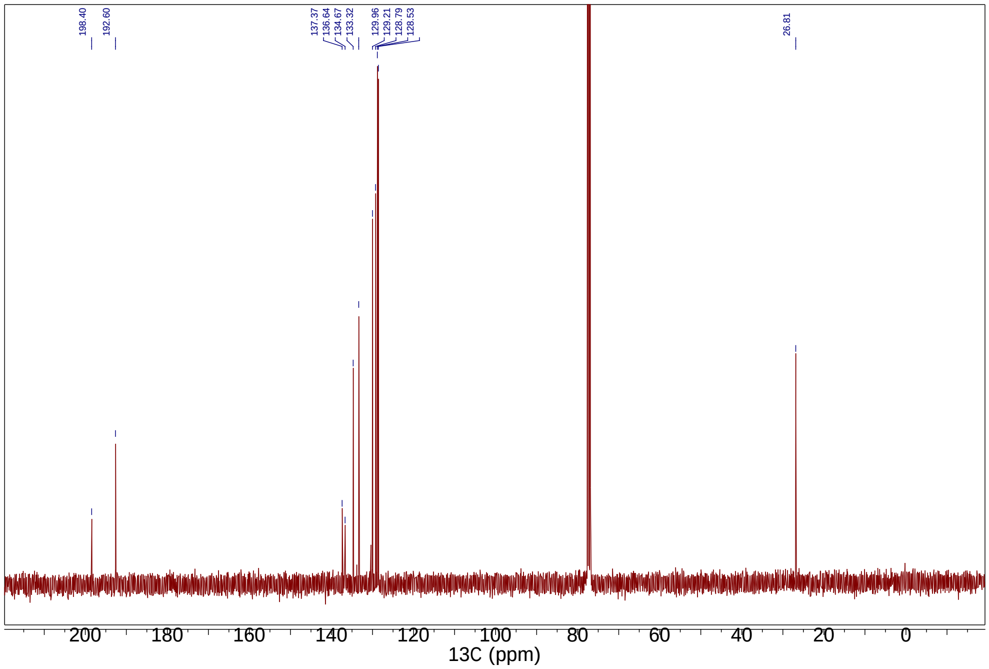

Figure S3B: Benzaldehyde and acetophenone standard
^{13}C NMR (101 MHz, $CDCl_3$) δ 192.60, 136.64, 134.67, 129.96, 129.21 (benzaldehyde); 198.40, 137.37, 133.32, 128.79, 128.53, 26.81 (acetophenone).

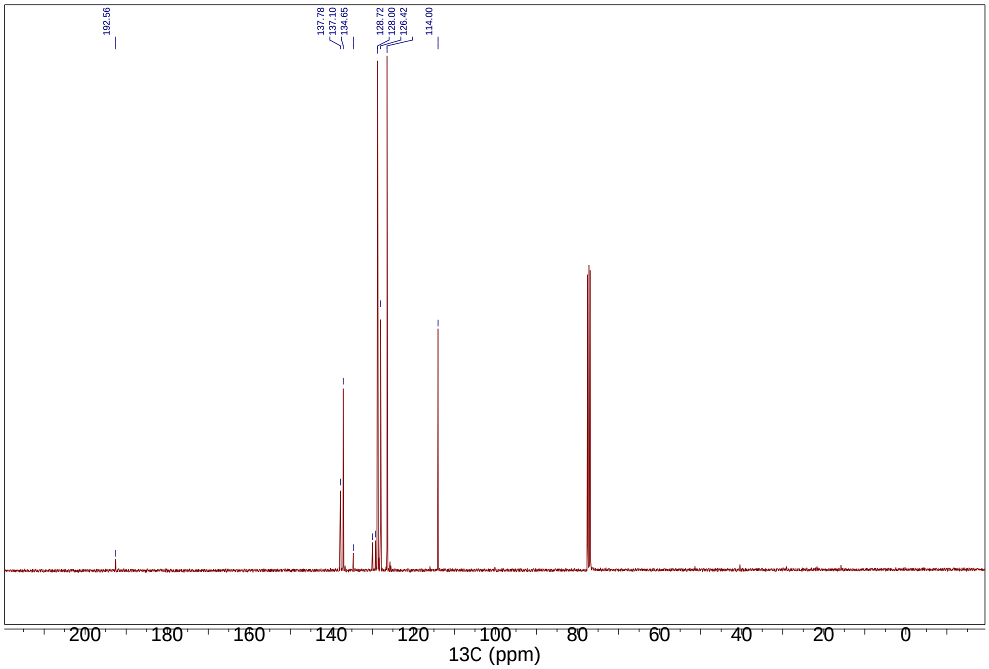

Figure S3C: Effluent gas trap solution from PS90 milled 360 min op.a
^{13}C NMR (101 MHz, $CDCl_3$) δ 192.56, 134.65, 129.95, 129.20 (benzaldehyde); 137.78, 137.10, 128.72, 128.00, 126.42, 114.00 (styrene).

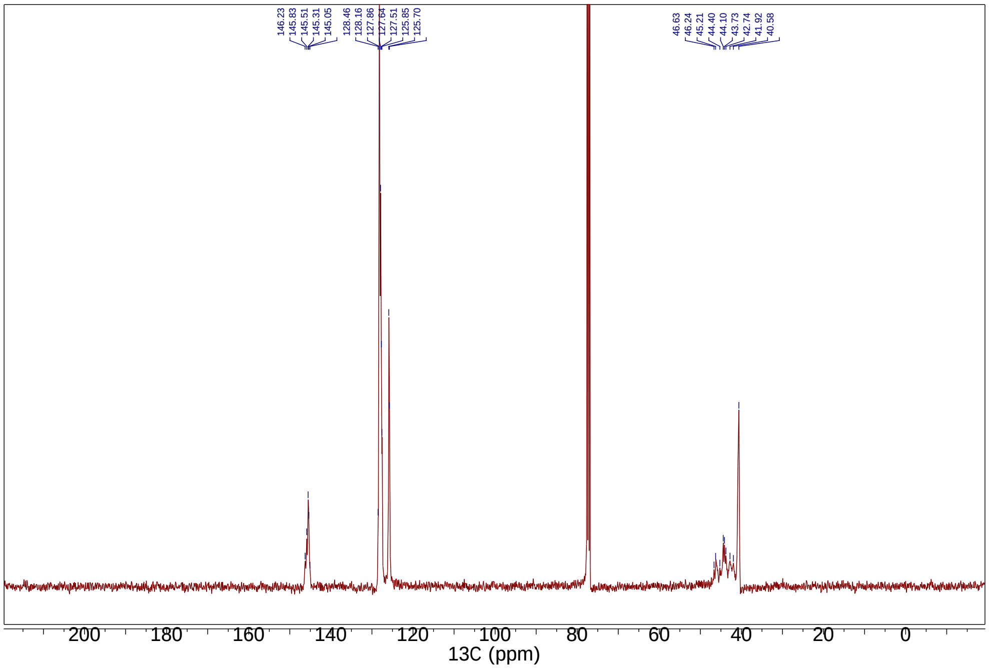

*Figure S3D: Unmilled PS90

^{13}C NMR (101 MHz, $CDCl_3$) δ 146.23, 145.83, 145.51, 145.31, 145.05, 128.46, 128.16, 127.86, 127.64, 127.51, 125.85, 125.70, 46.63, 46.24, 45.21, 44.40, 44.10, 43.73, 42.74, 41.92, 40.58.

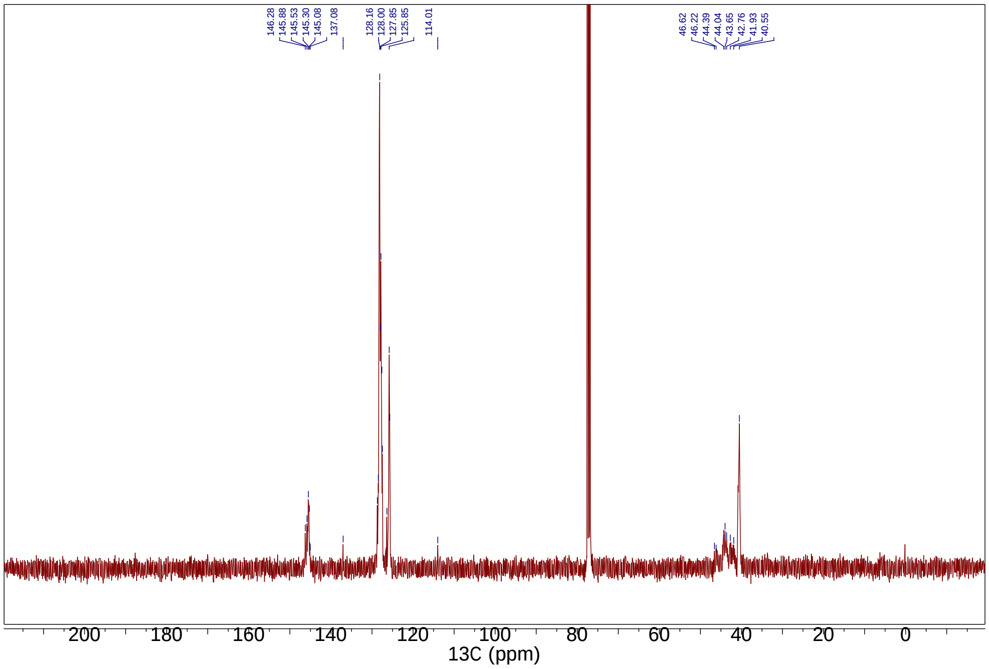

*Figure S3E: Solid residue PS90 milled 80 min cl.

^{13}C NMR (101 MHz, $CDCl_3$) δ 146.28, 145.88, 145.53, 145.30, 145.08, 128.48, 128.16, 127.85, 127.63, 127.52, 125.85, 125.70, 46.62, 46.22, 45.16, 44.39, 44.04, 43.65, 42.76, 41.93, 40.55 (PS90); 137.08, 128.72, 128.00, 126.41, 114.01 (styrene).

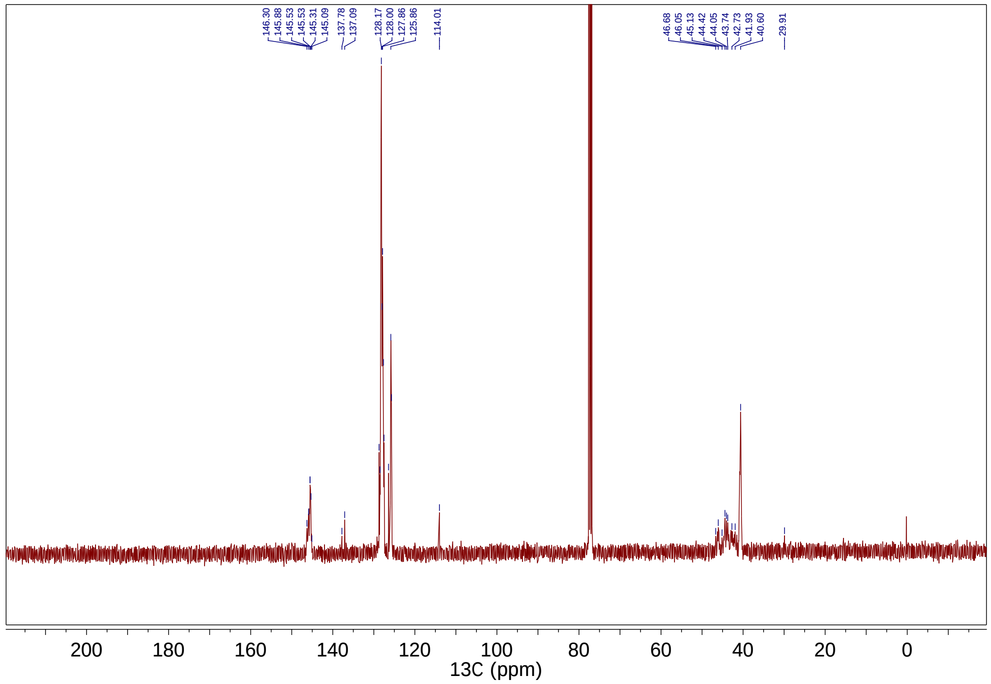

Figure S3F: Solid residue PS90 milled 80 min cl. with initial 40 mg styrene

^{13}C NMR (101 MHz, $CDCl_3$) δ 146.30, 145.88, 145.53, 145.31, 145.09, 128.48, 128.17, 127.86, 127.64, 127.51, 125.86, 125.70, 46.68, 46.05, 45.13, 44.42, 44.05, 43.74, 42.73, 41.93, 40.60, 29.91 (PS90); 137.78, 137.09, 128.72, 128.00, 126.41, 114.01 (styrene).

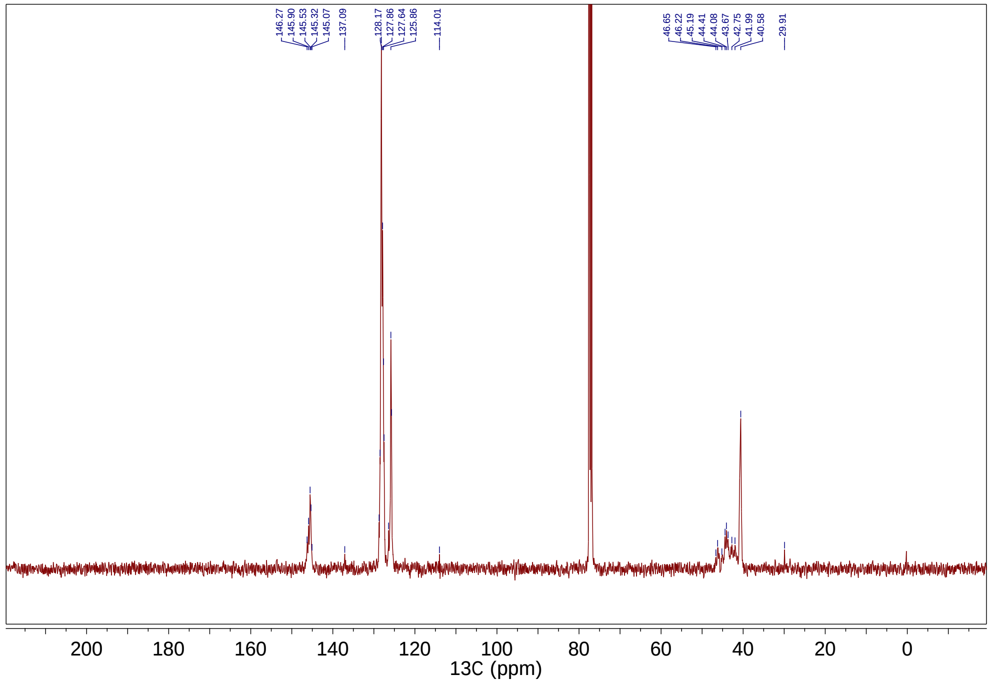

*Figure S3G: Solid residue PS90 milled 160 min cl.

^{13}C NMR (101 MHz, $CDCl_3$) δ 146.27, 145.90, 145.53, 145.32, 145.07, 128.48, 128.17, 127.86, 127.64, 127.52, 125.86, 125.70, 46.65, 46.22, 45.19, 44.41, 44.08, 43.67, 42.75, 41.99, 40.58, 29.91 (PS90); 137.09, 128.72, 126.41, 114.01 (styrene).

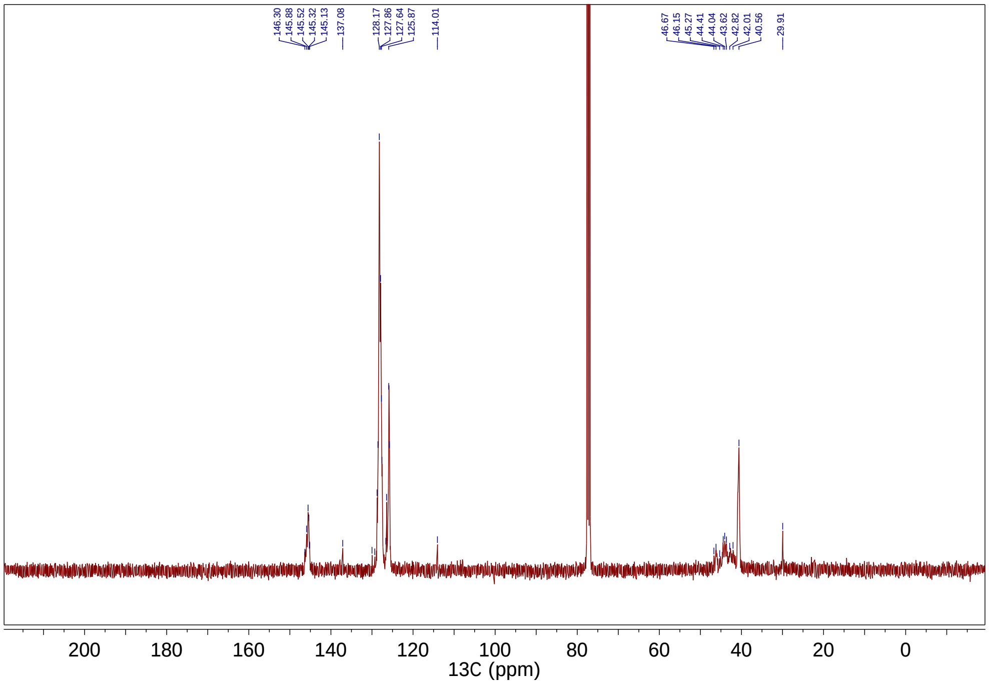

*Figure S3H: Solid residue PS90 milled 240 min cl.

^{13}C NMR (101 MHz, $CDCl_3$) δ 146.30, 145.88, 145.52, 145.32, 145.13, 128.50, 128.17, 127.86, 127.64, 127.52, 126.58, 125.87, 125.70, 46.67, 46.15, 45.27, 44.41, 44.04, 43.62, 42.82, 42.01, 40.56, 29.91 (PS90); 137.08, 128.72, 126.41, 114.01 (styrene); 129.95, 129.26 (benzaldehyde).

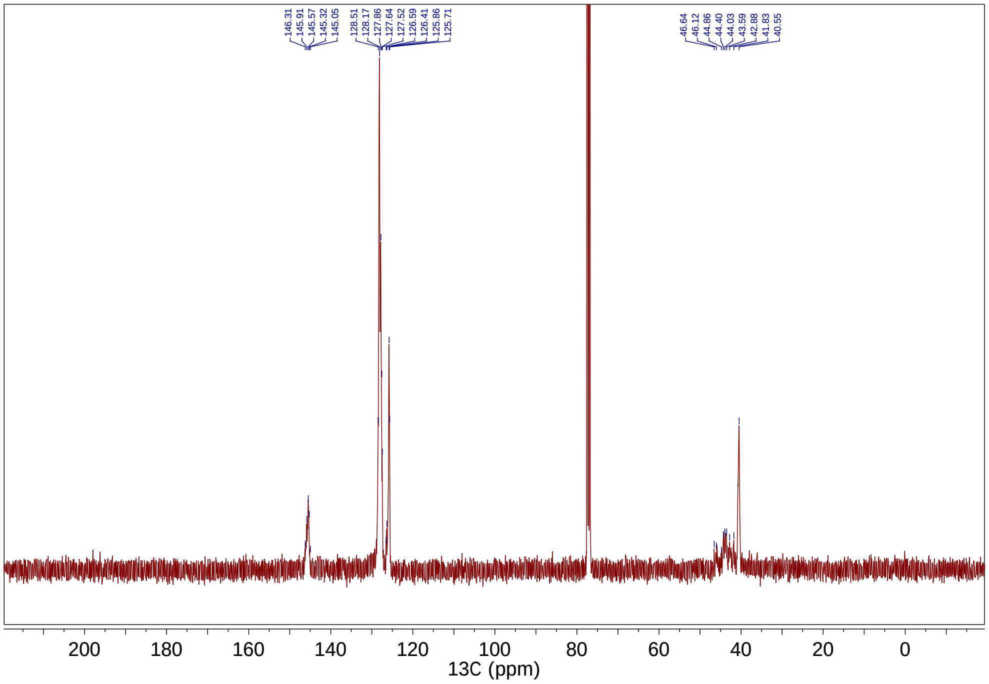

Figure S3I: Solid residue PS90 milled 240 min op.n

^{13}C NMR (101 MHz, $CDCl_3$) δ 146.31, 145.91, 145.57, 145.32, 145.05, 128.51, 128.17, 127.86, 127.64, 127.52, 126.59, 125.86, 125.71, 46.64, 46.12, 44.86, 44.40, 44.03, 43.59, 42.88, 41.83, 40.55 (PS90); 126.41 (styrene).

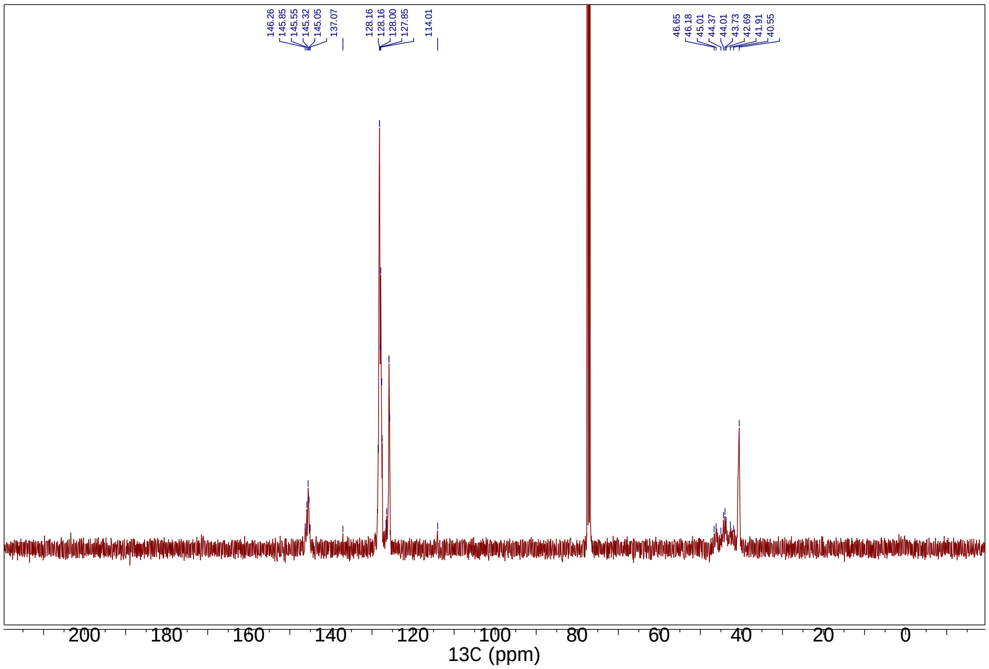

Figure S3J: Solid residue PS90 milled 240 min op.a
^{13}C NMR (101 MHz, $CDCl_3$) δ 146.26, 145.85, 145.55, 145.32, 145.05, 128.48, 128.16, 127.85, 127.64, 127.51, 126.59, 125.86, 125.70, 46.65, 46.18, 45.01, 44.37, 44.01, 43.73, 42.69, 41.91, 40.55 (PS90); 137.07, 128.00, 126.41, 114.01 (styrene).

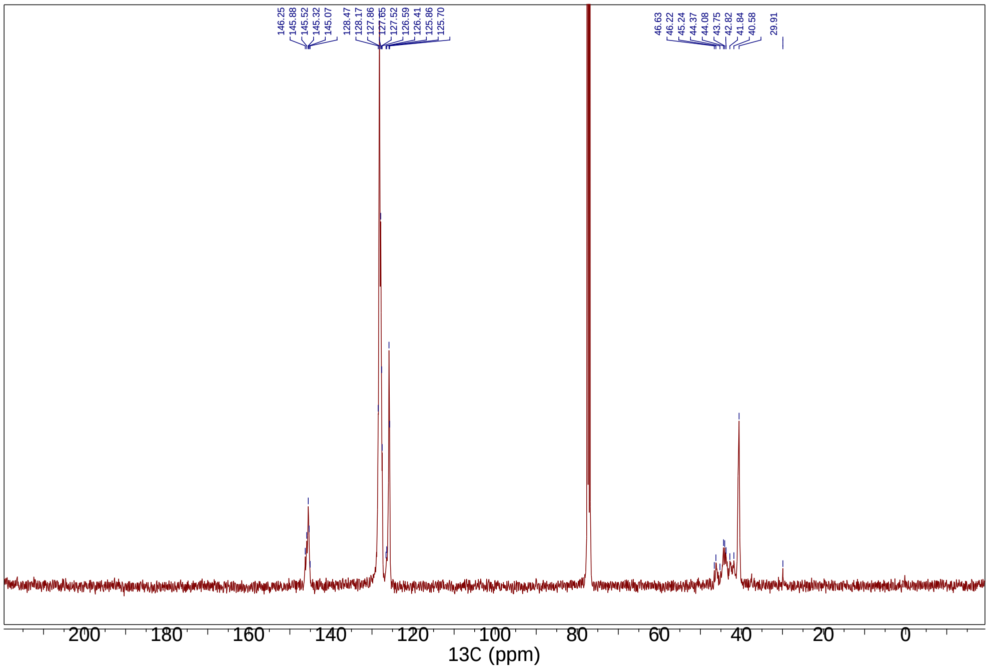

*Figure S3K: Solid residue PS90 milled 360 min op.a
^{13}C NMR (101 MHz, $CDCl_3$) δ 146.25, 145.88, 145.52, 145.32, 145.07, 128.47, 128.17, 127.86, 127.65, 127.52, 126.59, 125.86, 125.70, 46.63, 46.22, 45.24, 44.37, 44.08, 43.75, 42.82, 41.84, 40.58, 29.91 (PS90); 126.41 (styrene).

S.D2. ^{1}H spectra

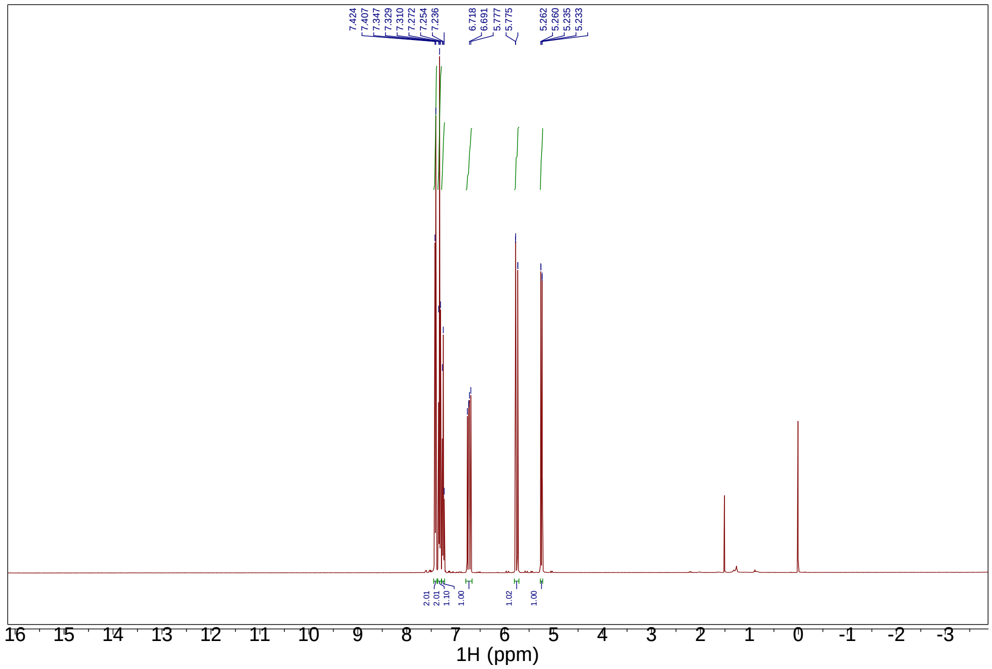

Figure S4A: Styrene standard
^{1}H NMR (400 MHz, $CDCl_3$) δ (7.42, 7.40), (7.34, 7.32, 7.30), (7.26, 7.25, 7.23), (6.75, 6.73, 6.71, 6.68), (5.77, 5.73), (5.25, 5.23).

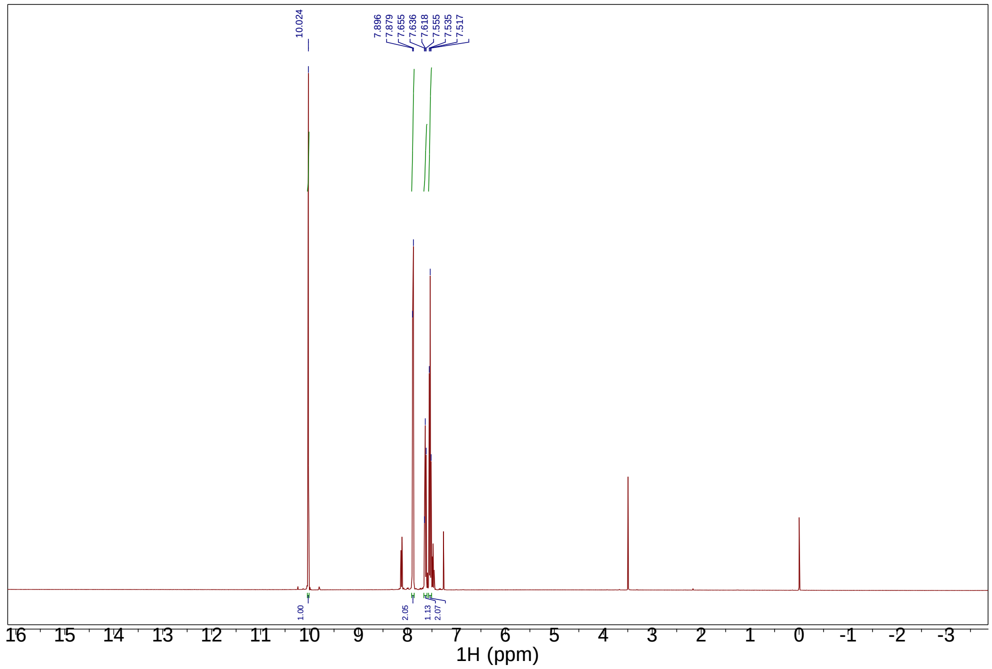

Figure S4B: Benzaldehyde standard
^{1}H NMR (400 MHz, $CDCl_3$) δ 10.03, (7.90, 7.88), (7.66, 7.64, 7.62), (7.56, 7.54, 7.52).

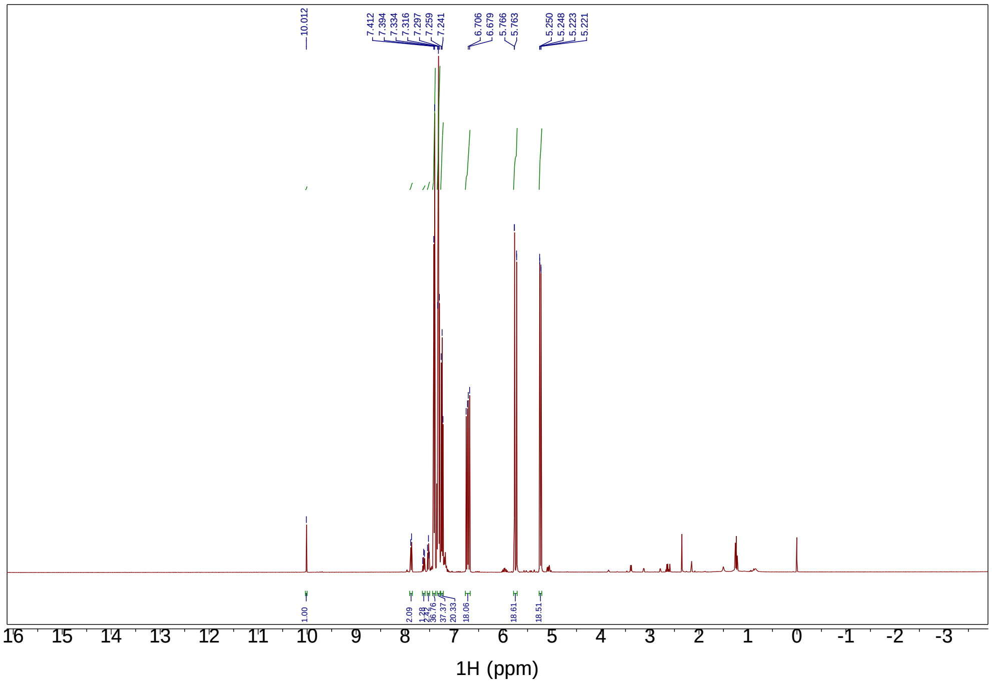

Figure S4C: Effluent gas trap solution from PS90 milled 360 min op.a
^{1}H NMR (400 MHz, $CDCl_3$) δ 10.01, (7.88, 7.86), (7.64, 7.62, 7.60), (7.54, 7.52, 7.50) (benzaldehyde); (7.41, 7.39), (7.33, 7.32, 7.30), (7.26, 7.24, 7.22), (6.75, 6.72, 6.71, 6.68), (5.76, 5.72), (5.24, 5.22) (styrene).

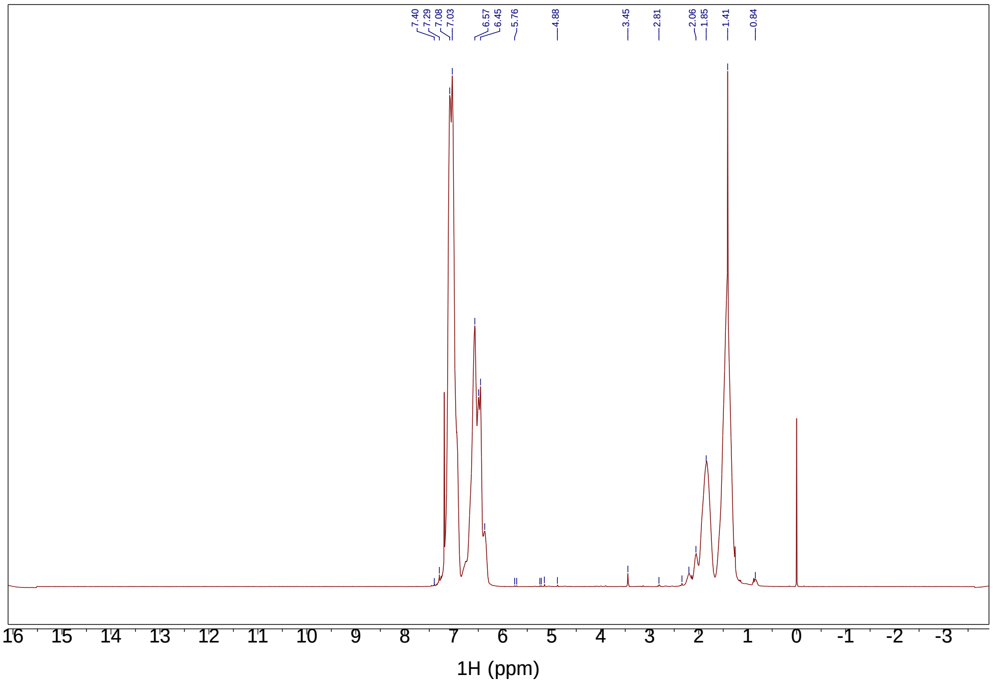

Figure S4D: Unmilled PS90
^{1}H NMR (400 MHz, $CDCl_3$) δ 7.40, 7.29 (styrene-like); 7.08, 7.03, 6.57, 6.49, 6.45, 6.37, 5.76, 5.72, 5.24, 5.21, 5.15, 4.88, 2.81, 2.34, 2.20, 2.06, 1.85, 1.41, 0.84 (PS90); 3.45 (methanol – impurity).

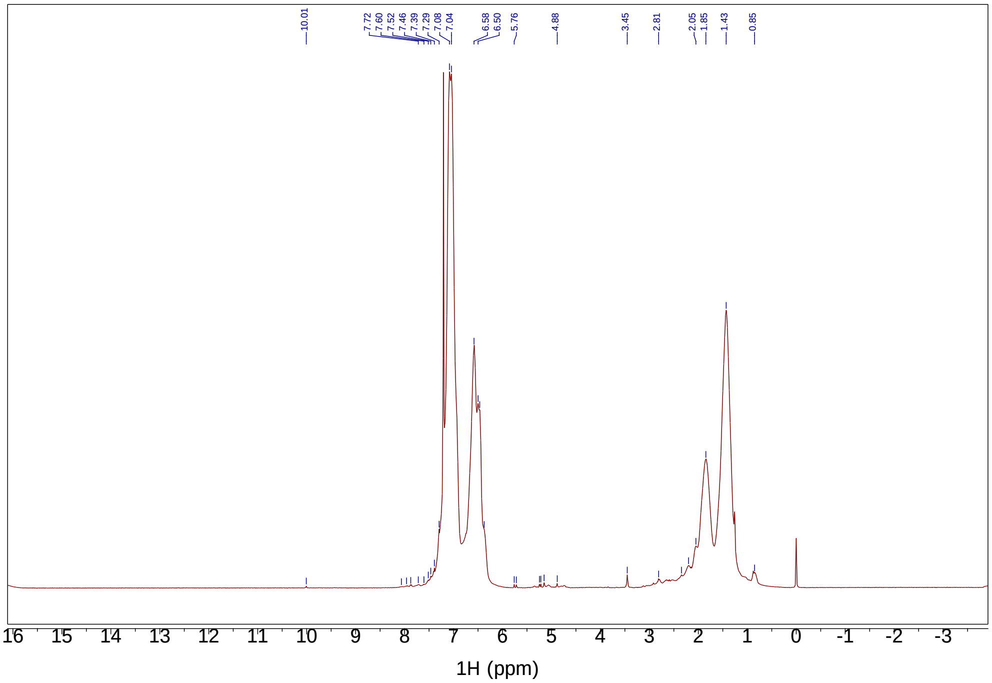

Figure S4E: Solid residue PS90 milled 360 min op.a

^{1}H NMR (400 MHz, $CDCl_3$) δ 10.01, 7.87, 7.60, 7.52 (benzaldehyde); 8.06, 7.96, 7.72, 7.46 (unassigned monomers); 7.39, 7.29 (styrene-like); 7.08, 7.04, 6.58, 6.50, 6.46, 6.37, 5.76, 5.72, 5.24, 5.22, 5.15, 4.88, 2.81, 2.34, 2.20, 2.05, 1.85, 1.43, 0.85 (PS90); 3.45 (methanol – impurity).

S.E. Attenuated Total Reflectance Fourier-transform Infrared Spectroscopy (ATR-FTIR)
The IR spectrum of pre-milled PS90 powder was measured with the Thermo Scientific™ Nicolet™ 8700 FTIR spectrometer equiped with Smart iTR™ Attenuated Total Reflectance (ATR) sampling unit with a diamond/ZnSe crystal lens.

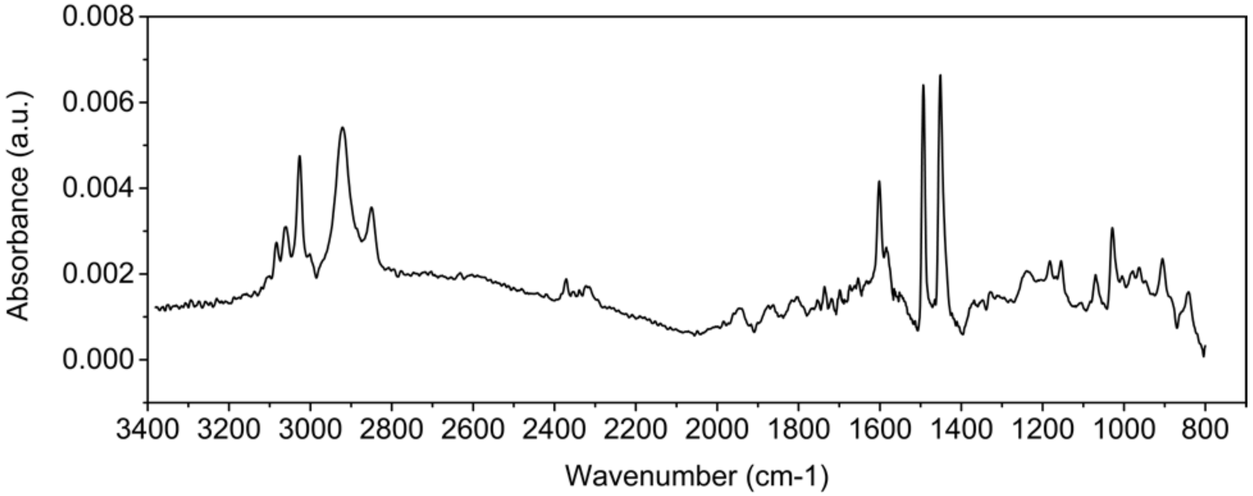

Figure S5: IR spectrum of PS90 starting material for experiments, pre-milled using the following specifications: 10 g of PS pellets and two 20 mm diameter stainless steel balls were loaded into a 50 mL Retsch steel reactor and milled on a Retsch MM400 shaker mill at 30 Hz for 4 minutes under ambient atmosphere.

S.F. Dynamic Light Scattering (DLS)
Particle size distributions of milled PS90 cl. residues were measured using a Wyatt Technology DynaPro NanoStar instrument. 25 mg of residue powder was dispersed in 50 mL of deionized water containing 100 μL of 1-butanol using a bath sonicator. 1 mL of this dispersion was dispensed into a PS cuvette for analysis.

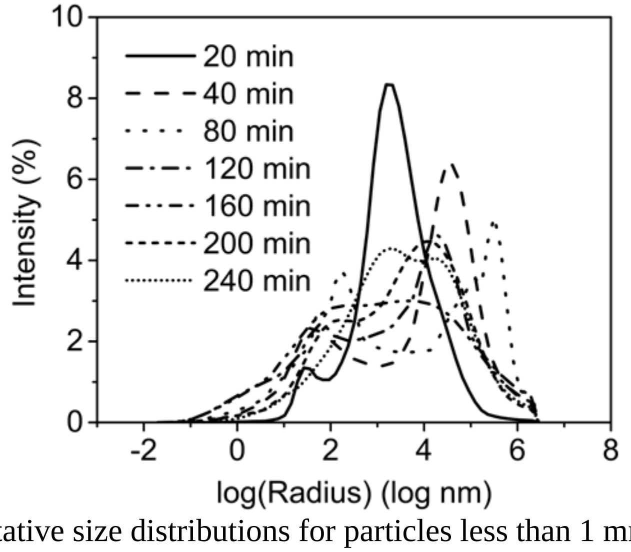

Figure S6: Representative size distributions for particles less than 1 mm for various milling times. For milling times of 20, 40 and 80 min., the distribution maximum occurs at lower radii because most of the particles in these samples were greater than the size detection limit of the instrument, thus the obtained distribution for these samples represent only a minority of the particles small enough to be detected.
